# Supplementary material for: Three Small Cysteine-Free Proteins (CFP1–3) Are Required for Insect-Pathogenic Lifestyle of Metarhizium robertsii
Source: J Fungi (Basel). 2022 Jun 6;8(6):606. doi: 10.3390/jof8060606 (PMC9224661; doi:10.3390/jof8060606)
Supplement: Supplementary file 1 [file jof-08-00606-s001.zip › jof-1726415-supplementary.pdf]

Supplementary Material for Journal of Fungi

Three Small Cysteine-Free Proteins (CFP1–3) Are Required for Insect-Pathogenic Lifestyle of *Metarhizium robertsii*

Ya-Ni Mou, Kang Ren, Sheng-Hua Ying, and Ming-Guang Feng *
(*Corresponding author. E-mail: mgfeng@zju.edu.cn)

Contents:

Figure S1. Generation and identification of *cfp1, cfp2 and cfp3* mutants in *M. robertsii*, p. 2
Table S1. Paired primers designed for manipulation of *cfp1, cfp2 and cfp3* in *M. robertsii*, p. 2
Table S2. Differentially expressed genes identified from the transcriptome of *M. robertsii* Δ*cfp1* versus WT strain, pp. 3-12
Table S3. GO analysis of differentially expressed genes in *M. robertsii* Δ*cfp1* versus WT strain, pp. 13-24
Table S4. Summation of GO function classes and terms enriched to *M. robertsii* Δ*cfp1* versus WT strain, pp. 25-27
Table S5. KEGG pathway analysis of differential expressed genes in *M. robertsii* Δ*cfp1* versus WT strain, pp. 28-30
Table S6. Summation of KEGG pathways enriched to *M. robertsii* Δ*cfp1* versus WT strain, p. 30
Table S7. Differentially expressed genes related to phenotypes and genome stability of *M. robertsii* Δ*cfp1* versus WT strain, pp. 31-33

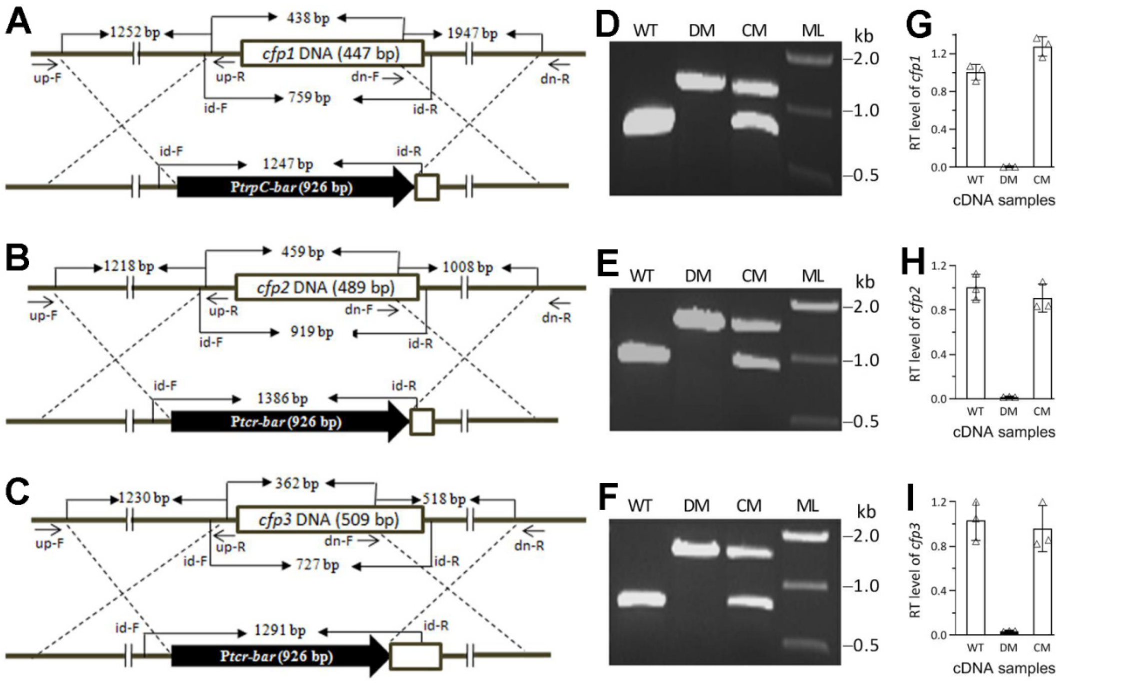

Figure S1. Generation and identification of *cfp1, cfp2 and cfp3* mutants in *M. robertsii*. (**A–C**) Schematic diagrams for the deletion strategies of *cfp1*, *cfp2* and *cfp3*. (**D–F**) The deletion mutants (DM) and complementation mutants (CM) of *cfp1*, *cfp2* and *cfp3* identified respectively by PCR analysis (ML, DNA molecular ladder) with paired primers (Table S1). The DNA fragments detected from WT, DM and CM indicate successful deletions of partial promoter and coding regions from *cfp1* (46+392 = 926+759–1247 = 438 bp), *cfp2* (75+384 = 926+919–1386 = 459 bp) and *cfp3* (16+346 = 926+727–1291 = 362 bp) as expected or shown in the diagrams, respectively. (**G–I**) Relative transcript (RT) levels of *cfp1*, *cfp2* and *cfp3* in the cDNA samples derived from the 3 d-old 1/4 SDAY cultures of targeted gene mutants with respect to the WT standard. Error bars: standard deviations from three cDNA samples derived from independent cultures of each strain.

Table S1. Paired primers designed for manipulation of *cfp1, cfp2 and cfp3* in *M. robertsii.*

Primers	Paired sequences (5 3)*	Purpose
upCfp1-F/R	ACGAGCTGTACAAGTAACCCGGGTGGCAAGCTCCTCTGATC / TGGCTGCAGGTCGACGGATCCGGTGTTCTTTATGGGAAGTTAT	Cloning *cfp1* 5'-end (1252 bp)
dnCfp1-F/R	GACCCATGGCTCGAGTCTAGATAATCAAGCCTATTGGAACA / GGTGGTGGTGGCTAGCGTTAACGCACCACGAACCTCTACC	Cloning *cfp1* 3'-end (1947 bp)
flCfp1-F/R	ATCCGTCGACCTGCAGCCAAGCTTGCTTCATCTCATCGTCCAA / ACACTAGTCAGATCTTCTAGTGTTGTCAACCTCGTGCTCCT	Cloning full-length *cfp1* (4194 bp) for complementation
upCfp2-F/R	ACGAGCTGTACAAGTAACCCGGGGCGTCTGTTATCGGGTTG / TGGCTGCAGGTCGACGGATCCTGCGGTGAGTGTTTGATTT	Cloning *cfp2* 5'-end (1218 bp)
dnCfp2-F/R	GACCCATGGCTCGAGTCTAGAGGGACGAACAAGGCTAAT / GGTGGTGGTGGCTAGCGTTAACCAACATCCATCGCTACGC	Cloning *cfp2* 3'-end (1008 bp)
flCfp2-F/R	ATCCGTCGACCTGCAGCCAAGCTTCGGACCTGATGGAACCTG / ACACTAGTCAGATCTTCTAGTGTCGCTGATGCTCGCACAAA	Cloning full-length *cfp2* (4078 bp) for complementation
upCfp3-F/R	ACGAGCTGTACAAGTAACCCGGGCAAAGATTGTGAGCCTGAA / TGGCTGCAGGTCGACGGATCCGGAAGTGTTGGAAGTAGATGA	Cloning *cfp3* 5'-end (1230 bp)
dnCfp3-F/R	GACCCATGGCTCGAGTCTAGAGCCACTTCGTCTGGACAGT / GGTGGTGGTGGCTAGCGTTAACGCCCTCCTCCGAGATAGTT	Cloning *cfp3* 3'-end (1097 bp)
flCfp3-F/R	ATCCGTCGACCTGCAGCCAAGCTTACGACGATCTTGCACCTG / ACACTAGTCAGATCTTCTAGTGTCCGTTCCTATGATATGGC	Cloning full-length *cfp3* (2071 bp) for complementation
pCfp1-F/R	ACCACCAGAGTTTCCACG / TCACCAAGACCTCGCAAT	PCR detecting *cfp1*
pCfp2-F/R	GCCGGTAGGTGCTCTTAA / AGCGACTGTGGCTTCATC	PCR detecting *cfp2*
pCfp3-F/R	TGTCTCCCAAACAACTCAAC/ GAACTCCATACAACGGCTAT	PCR detecting *cfp3*
qCfp1-F/R	AAAACGTTGGCAAGCTCAAT / ATTCGTCCACCACGACTTTC	qPCR detecting *cfp1*
qCfp2-F/R	TTCTTCTTGCCGTCTTTTCC / GCTGAGCTGCTCCTTCAACT	qPCR detecting *cfp2*
qCfp3-F/R	CCAGACTTCAGCCGTAAAGG / ACATCTTTTGCCTCCGTTTG	qPCR detecting *cfp3*

* Underlined regions denote introduced cleavage sites of two pairs of restriction enzymes (XmaI/BamHI and XbaI/HpaI) for homologous recombination of 5' and 3' fragments separated by *bar* marker for targeted *cfp* deletion or the recognition fragments to exchange for the gateway fragment.

Table S2. Differentially expressed genes identified from the transcriptome of *M. robertsii* Δ*cfp1* versus WT strain.

gene_id	gene_name	transcript_id	$\log_2 R$	*q*-value	Annotation
19254709	MAA_00423	XM_007818421.2	-16.96	1.99E-21	Ribonuclease H-like protein
19257485	MAA_03199	XM_007821197.1	-15.31	1.80E-11	hypothetical protein MAA_03199
19262961	MAA_08675	XM_007826673.1	-14.89	5.61E-05	hypothetical protein MAA_08675
23632372	MAA_10923	XM_011412869.1	-14.77	4.07E-09	Ankyrin repeat-containing domain protein
19260937	MAA_06651	XM_007824649.1	-14.01	1.05E-04	hypothetical protein MAA_06651
19254669	MAA_00383	XM_007818381.2	-13.98	9.65E-10	Ribonuclease H-like protein
23633117	MAA_11669	XM_011412584.1	-13.77	7.64E-12	hypothetical protein MAA_11669
23632348	MAA_10899	XM_011412845.1	-13.25	1.28E-04	Proteinase inhibitor I78
19255569	MAA_01283	XM_007819281.2	-12.64	1.02E-07	Ribonuclease H-like protein
23633245	MAA_11797	XM_011412985.1	-12.43	2.09E-06	hypothetical protein MAA_11797
19261241	MAA_06955	XM_007824953.1	-11.71	1.04E-03	lipase 2
19262574	MAA_08288	XM_007826286.1	-11.53	4.27E-02	Cyclin-like F-box
19264575	MAA_10289	XM_007828287.1	-11.27	3.19E-03	periplasmic binding protein
19264125	MAA_09839	XM_007827837.1	-10.97	2.54E-02	hypothetical protein MAA_09839
19264807	MAA_10521	XM_007828519.2	-10.75	2.51E-02	zinc knuckle
19254642	MAA_00356	XM_007818354.1	-10.67	4.28E-02	glycosyl transferase
19264862	MAA_10576	XM_007828574.1	-10.62	1.25E-02	Uncharacterized protein MAA_10576
19255828	MAA_01542	XM_007819540.2	-10.54	4.86E-02	hypothetical protein MAA_01542
23633036	MAA_11588	XM_011412503.1	-9.96	3.33E-04	SET domain protein
19261030	MAA_06744	XM_007824742.1	-9.56	1.19E-03	lysergyl peptide synthetase subunit 3 LPS3
19262985	MAA_08699	XM_007826697.1	-8.86	4.03E-02	Beta-ketoacyl synthase
19254385	MAA_00099	XM_007818097.1	-8.54	1.79E-10	hypothetical protein MAA_00099
19261770	MAA_07484	XM_007825482.1	-7.95	3.74E-14	peptidase alpha-lytic pro domain protein
19263004	MAA_08718	XM_007826716.1	-6.63	4.34E-10	Peptidase S8, subtilisin, Asp-active site protein
19261481	MAA_07195	XM_007825193.2	-5.86	5.55E-08	protein-tyrosine phosphatase
19263490	MAA_09204	XM_007827202.1	-5.66	1.11E-18	Concanavalin A-like lectin/glucanase
19262600	MAA_08314	XM_007826312.2	-5.35	1.77E-03	ATPase
19263823	MAA_09537	XM_007827535.1	-5.23	1.69E-04	MFS1 family protein
19262598	MAA_08312	XM_007826310.1	-5.18	1.21E-43	cell wall protein
19259182	MAA_04896	XM_007822992.1	-5.06	3.47E-10	regulatory P domain-containing protein
19260861	MAA_06575	XM_007824573.1	-4.95	7.68E-04	Beta-ketoacyl synthase
19255257	MAA_00971	XM_007818969.2	-4.81	4.46E-02	Aromatic prenyltransferase, DMATS type
19264559	MAA_10273	XM_007828271.1	-4.71	8.64E-04	Asparaginase/glutaminase
19264560	MAA_10274	XM_007828272.1	-4.58	1.41E-03	TAM domain methyltransferase
19259961	MAA_05675	XM_007823673.1	-4.48	4.98E-44	alkaline serine protease P32
19262599	MAA_08313	XM_007826311.2	-4.41	6.17E-22	phytanoyl-CoA dioxygenase family protein
19262115	MAA_07829	XM_007825827.2	-4.38	1.12E-11	WD40 repeat-like-containing domain protein
19259268	MAA_04982	XM_007822493.1	-4.36	3.19E-09	protein-arginine deiminase type-4
23632349	MAA_10900	XM_011412846.1	-4.32	9.22E-35	hypothetical protein MAA_10900
19263595	MAA_09309	XM_007827307.1	-4.24	2.26E-56	hypothetical protein MAA_09309
19257629	MAA_03343	XM_007821341.1	-4.12	7.13E-05	Gram-positive signal peptide protein, YSIRK family
19255283	MAA_00997	XM_007818995.1	-4.09	2.01E-12	peptidase family M28
19255619	MAA_01333	XM_007819331.1	-4.06	1.89E-20	carboxypeptidase, partial
23632927	MAA_11479	XM_011413473.1	-4.02	3.54E-02	hypothetical protein MAA_11479
23633146	MAA_11698	XM_011412613.1	-3.98	4.99E-04	hypothetical protein MAA_11698
23633214	MAA_11766	XM_011412804.1	-3.86	1.16E-02	hypothetical protein MAA_11766
19257526	MAA_03240	XM_007821238.1	-3.79	2.33E-02	hypothetical protein MAA_03240
23632468	MAA_11020	XM_011412965.1	-3.77	2.95E-02	peptidase S1 domain protein
19264332	MAA_10046	XM_007828044.2	-3.68	8.84E-39	Aspartic acid decarboxylase, DtxS4
19260466	MAA_06180	XM_007824178.2	-3.64	8.90E-09	peroxidase manganese-dependent 1
19257561	MAA_03275	XM_007821273.2	-3.64	3.05E-02	transferase
19262048	MAA_07762	XM_007825760.1	-3.64	3.04E-04	hypothetical protein MAA_07762
19263074	MAA_08788	XM_007826786.2	-3.60	2.98E-05	ankyrin repeat-containing protein
19262592	MAA_08306	XM_007826304.1	-3.45	1.96E-04	Ankyrin repeat protein
19262751	MAA_08465	XM_007826463.2	-3.33	1.09E-02	hypothetical protein MAA_08465
19256231	MAA_01945	XM_007819943.1	-3.30	1.09E-25	fungalysin metallopeptidase
19262135	MAA_07849	XM_007825847.1	-3.25	3.96E-11	protein-arginine deiminase type-4
19264708	MAA_10422	XM_007828420.1	-3.24	2.86E-02	hypothetical protein MAA_10422

19264334	MAA_10048	XM_007828046.2	-3.21	7.70E-27	abc transporter
19261383	MAA_07097	XM_007825095.2	-3.19	5.20E-03	C2H2 transcription factor
19257889	MAA_03603	XM_007821601.1	-3.18	4.47E-25	PH domain-containing protein
19264333	MAA_10047	XM_007828045.2	-3.17	1.21E-25	V-type ATPase
19263838	MAA_09552	XM_007827550.1	-3.11	2.85E-02	FAD-binding, type 2
23633156	MAA_11708	XM_011412623.1	-3.09	3.15E-04	hypothetical protein X797_011588
19263878	MAA_09592	XM_007827590.1	-3.08	2.12E-25	Peptidase S1/S6, chymotrypsin/Hap
19260495	MAA_06209	XM_007824207.1	-3.07	2.13E-10	Concanavalin A-like lectin/glucanase
19255716	MAA_01430	XM_007819428.1	-2.98	1.99E-13	Peptidase A1
23632897	MAA_11449	XM_011413443.1	-2.96	2.28E-17	Ankyrin repeat-containing domain protein
19257630	MAA_03344	XM_007821342.1	-2.92	1.61E-02	ankyrin repeat containing protein
19255921	MAA_01635	XM_007819633.1	-2.92	3.44E-04	integral membrane protein pth11
19262278	MAA_07992	XM_007825990.1	-2.83	6.79E-16	proline rich protein 5MeD
19254665	MAA_00379	XM_007818377.2	-2.77	2.53E-02	tlh5
19263667	MAA_09381	XM_007827379.1	-2.75	9.32E-09	protoporphyrinogen oxidase
19263325	MAA_09039	XM_007827037.2	-2.73	4.40E-06	hydantoinase
19259842	MAA_05556	XM_007823554.1	-2.72	2.47E-03	potassium/sodium efflux P-type ATPase
19264331	MAA_10045	XM_007828043.2	-2.72	6.37E-26	Aldo/keto reductase, DtxS3
19262512	MAA_08226	XM_007826224.1	-2.72	2.17E-06	Peptidase aspartic, active site protein
19263782	MAA_09496	XM_007827494.1	-2.70	3.60E-07	hypothetical protein MAA_09496
19263851	MAA_09565	XM_007827563.2	-2.70	5.30E-16	Metallophosphoesterase
19256710	MAA_02424	XM_007820422.2	-2.67	1.10E-02	hypothetical protein X797_000808
23632304	MAA_10855	XM_011412774.1	-2.66	1.49E-05	hypothetical protein MAA_10855
19263200	MAA_08914	XM_007826912.1	-2.62	1.14E-02	VrtL
19261160	MAA_06874	XM_007824872.2	-2.59	2.30E-11	Necrosis inducing
19264969	MAA_10685	XM_011412403.1	-2.56	6.46E-03	hypothetical protein MAA_10685
19263572	MAA_09286	XM_007827284.1	-2.51	8.55E-04	hypothetical protein MAA_09286
23632924	MAA_11476	XM_011413470.1	-2.51	3.28E-02	hypothetical protein MAA_11476
19262976	MAA_08690	XM_007826688.2	-2.48	2.44E-02	hypothetical protein MAA_08690
19262950	MAA_08664	XM_007826662.1	-2.47	6.37E-03	hypothetical protein MAA_08664
19259811	MAA_05525	XM_007823523.1	-2.46	2.67E-07	endo-1,3(4)-beta-glucanase
19259506	MAA_05220	XM_007823218.1	-2.46	1.06E-15	hypothetical protein MAA_05220
19256036	MAA_01750	XM_007819748.1	-2.42	7.05E-08	bacterial-type extracellular deoxyribonuclease
19257223	MAA_02937	XM_007820935.1	-2.41	5.67E-05	hypothetical protein MAA_02937
19257548	MAA_03262	XM_007821260.1	-2.40	3.01E-10	Peptidase S1/S6, chymotrypsin/Hap
19262575	MAA_08289	XM_007826287.1	-2.35	1.96E-03	cell surface protein (Mas1)
19257525	MAA_03239	XM_007821237.2	-2.33	6.22E-06	Beta-ketoacyl synthase
19259759	MAA_05473	XM_007823471.1	-2.32	7.02E-05	siderophore iron transporter mirB
23632915	MAA_11467	XM_011413461.1	-2.32	1.69E-13	hypothetical protein MAA_11467
19262601	MAA_08315	XM_007826313.1	-2.30	1.80E-03	Glycoside hydrolase, chitinase active site protein
19261161	MAA_06875	XM_007824873.1	-2.29	1.92E-05	Monooxygenase, FAD-binding protein
23633287	MAA_11839	XM_011413513.1	-2.28	1.09E-02	hypothetical protein MAA_11839
19263527	MAA_09241	XM_007827239.1	-2.28	2.55E-02	cysteine-rich secretory protein
19262202	MAA_07916	XM_007825914.2	-2.26	2.10E-02	major facilitator superfamily transporter
19263547	MAA_09261	XM_007827259.1	-2.25	1.71E-02	Regulator of G protein signaling superfamily
19264800	MAA_10514	XM_007828512.1	-2.24	1.16E-03	hypothetical protein MAA_10514
23632640	MAA_11192	XM_011413172.1	-2.20	4.26E-03	ankyrin repeat protein
19264670	MAA_10384	XM_007828382.1	-2.20	2.66E-09	dynamin family protein
19262060	MAA_07774	XM_007825772.2	-2.19	1.16E-02	ankyrin protein
19262606	MAA_08320	XM_007826318.2	-2.18	9.18E-09	LAS seventeen-binding protein
19264754	MAA_10468	XM_007828466.1	-2.18	1.35E-03	catalytic protein
19255215	MAA_00929	XM_007818927.2	-2.17	3.22E-07	Ribonuclease H-like protein
19254299	MAA_00013	XM_007818011.1	-2.17	2.08E-04	lipase, class 3
19263566	MAA_09280	XM_007827278.1	-2.16	9.04E-03	hypothetical protein MAA_09280
19255798	MAA_01512	XM_007819510.2	-2.15	1.08E-07	Cytochrome P450 CYP5148B3
19262357	MAA_08071	XM_007826069.1	-2.14	2.61E-05	hypothetical protein MAA_08071
19256264	MAA_01978	XM_007819976.1	-2.14	1.82E-02	hypothetical protein MAA_01978
19264234	MAA_09948	XM_007827946.1	-2.13	3.17E-02	nitroreductase protein
19261774	MAA_07488	XM_007825486.2	-2.11	3.47E-10	exo-1,3-beta-D-glucanase
19259153	MAA_04867	XM_007822963.2	-2.08	1.21E-02	Pyridine nucleotide-disulfide oxidoreductase, NAD-binding domain
19262411	MAA_08125	XM_007826123.1	-2.05	1.80E-02	hypothetical protein MAA_08125

19263916	MAA_09630	XM_007827628.1	-2.03	2.15E-21	alpha-N-acetylgalactosaminidase
19261773	MAA_07487	XM_007825485.2	-2.03	1.23E-06	hypothetical protein MAA_07487
19259775	MAA_05489	XM_007823487.1	-2.03	4.55E-02	Oxoglutarate/iron-dependent oxygenase
19263663	MAA_09377	XM_007827375.1	-2.01	1.86E-07	SPFH domain/Band 7 family protein
19262292	MAA_08006	XM_007826004.1	-1.99	1.48E-16	hypothetical protein MAA_08006
19259894	MAA_05608	XM_007823606.1	-1.98	9.09E-03	hypothetical protein MAA_05608
19254732	MAA_00446	XM_007818444.1	-1.97	4.15E-05	NADPH dehydrogenase
19259729	MAA_05443	XM_007823441.1	-1.96	2.64E-07	siderophore biosynthesis protein
19260857	MAA_06571	XM_007824569.1	-1.95	2.42E-02	NAD(P)-binding domain protein
19264680	MAA_10394	XM_007828392.1	-1.93	4.18E-02	hypothetical protein MAA_10394
19256668	MAA_02382	XM_007820380.2	-1.91	3.05E-09	Oligopeptide transporter OPT superfamily
19258919	MAA_04633	XM_007822729.2	-1.90	3.25E-02	hypothetical protein MAA_04633
19260674	MAA_06388	XM_007824386.2	-1.90	3.36E-12	Cd2+/Zn2+ transporter protein
19258334	MAA_04048	XM_007822046.2	-1.90	1.77E-03	major facilitator superfamily transporter
19264673	MAA_10387	XM_007828385.2	-1.89	4.30E-06	ZIP Zinc transporter
19261014	MAA_06728	XM_007824726.1	-1.89	5.86E-04	hypothetical protein MAA_06728
19264518	MAA_10232	XM_007828230.2	-1.88	2.63E-03	ankyrin repeat containing protein
19256072	MAA_01786	XM_007819784.2	-1.88	9.29E-05	hypothetical protein MAA_01786
19256824	MAA_02538	XM_007820536.1	-1.87	1.16E-06	hypothetical protein MAA_02538
19256825	MAA_02539	XM_007820537.1	-1.86	6.22E-06	cell surface protein (Mas1)
19263053	MAA_08767	XM_007826765.1	-1.85	3.64E-10	hypothetical protein MAA_08767
19255104	MAA_00818	XM_007818816.2	-1.85	2.94E-03	hypothetical protein X797_004960
19261648	MAA_07362	XM_007825360.1	-1.85	9.81E-04	RNA-directed RNA polymerase (Sad-1)
19254399	MAA_00113	XM_007818111.1	-1.84	3.80E-04	hypothetical protein MAA_00113
19259726	MAA_05440	XM_007823438.1	-1.83	7.89E-08	Phosphate transporter
19257585	MAA_03299	XM_007821297.1	-1.82	2.61E-07	hypothetical protein MAA_03299
19257527	MAA_03241	XM_007821239.2	-1.82	2.52E-02	glutamyl-tRNA(Gln) amidotransferase
19262022	MAA_07736	XM_007825734.1	-1.81	2.66E-02	Carbohydrate-binding WSC
19261096	MAA_06810	XM_007824808.1	-1.81	1.48E-06	FK506 suppressor Sfk1
19263304	MAA_09018	XM_007827016.2	-1.80	1.84E-04	Glycoside hydrolase, subgroup, catalytic core
19264330	MAA_10044	XM_007828042.2	-1.78	1.10E-19	Cytochrome P450, DtxS2
19263051	MAA_08765	XM_007826763.1	-1.78	7.74E-16	sugar transporter-like protein
19261778	MAA_07492	XM_007825490.1	-1.78	3.68E-03	c6 zinc finger domain containing protein
19263736	MAA_09450	XM_007827448.1	-1.76	3.24E-06	proteinrelated to ser/arg-related nuclear matrix protein
19264203	MAA_09917	XM_007827915.1	-1.74	2.98E-06	Cellular retinaldehyde-binding/triple function
23632201	MAA_10752	XM_011412470.1	-1.74	1.15E-02	triacylglycerol lipase-like protein
23633230	MAA_11782	XM_011412820.1	-1.74	8.65E-04	phosphoribosyl transferase domain protein
19261006	MAA_06720	XM_007824718.1	-1.74	2.63E-03	hypothetical protein MAA_06720
23633221	MAA_11773	XM_011412811.1	-1.72	1.67E-03	hypothetical protein MAA_11773
23633169	MAA_11721	XM_011412636.1	-1.71	1.69E-04	hypothetical protein MAA_11721
19254338	MAA_00052	XM_007818050.2	-1.71	1.05E-02	Nucleoside phosphorylase domain protein
19256493	MAA_02207	XM_007820205.1	-1.71	5.76E-04	carbohydrate esterase family 9 protein
19256663	MAA_02377	XM_007820375.1	-1.71	2.94E-06	ATP-binding cassette transporter ifT1
19262987	MAA_08701	XM_007826699.1	-1.69	8.60E-07	subtilisin-like serine protease precursor
19254288	MAA_00002	XM_007818000.1	-1.67	4.37E-05	hypothetical protein MAA_00002
19255591	MAA_01305	XM_007819303.2	-1.67	2.01E-09	hypothetical protein MAA_01305
19263800	MAA_09514	XM_007827512.2	-1.67	1.82E-03	Cytochrome P450 CYP58S1
19263052	MAA_08766	XM_007826764.1	-1.67	4.03E-11	maltase
19254713	MAA_00427	XM_007818425.1	-1.66	6.67E-04	hypothetical protein MAA_00427
19263326	MAA_09040	XM_007827038.2	-1.66	5.01E-03	NCS1 nucleoside transporter family protein
19259418	MAA_05132	XM_007823130.1	-1.66	2.26E-03	NAD(P)-binding protein
23632583	MAA_11135	XM_011413101.1	-1.65	1.82E-04	hypothetical protein X797_004552
19255592	MAA_01306	XM_007819304.1	-1.64	5.08E-04	protein kinase-like domain protein
19264428	MAA_10142	XM_007828140.1	-1.64	9.04E-03	Zn(2)-C6 fungal-type DNA-binding domain protein
19264998	MAA_10714	XM_011412432.1	-1.63	1.95E-03	C6 transcription factor
19258271	MAA_03985	XM_007821983.2	-1.63	1.17E-03	hypothetical protein MAA_03985
19264281	MAA_09995	XM_007827993.1	-1.63	9.51E-07	major facilitator superfamily transporter
19255540	MAA_01254	XM_007819252.1	-1.63	1.69E-04	hypothetical protein X797_005849
19263305	MAA_09019	XM_007827017.1	-1.62	7.26E-04	tri14-like protein
19263058	MAA_08772	XM_007826770.2	-1.62	8.43E-03	hypothetical protein MAA_08772
19255287	MAA_01001	XM_007818999.1	-1.61	9.33E-05	GCN5-related N-acetyltransferase (GNAT) domain protein

19257988	MAA_03702	XM_007821700.2	-1.61	1.80E-04	transcription factor RfeG
19261793	MAA_07507	XM_007825505.1	-1.59	1.67E-03	Pyoverdine biosynthesis
19263623	MAA_09337	XM_007827335.1	-1.58	8.09E-04	glyoxalase/bleomycin resistance protein/dioxygenase
23632692	MAA_11244	XM_011413224.1	-1.58	3.22E-07	hypothetical protein MAA_11244
23632734	MAA_11286	XM_011413266.1	-1.57	2.23E-04	nacht nucleoside triphosphatase
19264674	MAA_10388	XM_007828386.1	-1.57	1.07E-05	Glyoxalase/bleomycin resistance protein/dioxygenase
19260324	MAA_06038	XM_007824036.1	-1.57	2.17E-05	pH regulatory protein mrPacC
19261285	MAA_06999	XM_007824997.1	-1.56	2.04E-03	major facilitator superfamily transporter
19259509	MAA_05223	XM_007823221.1	-1.56	5.19E-03	CAS1 protein
19262506	MAA_08220	XM_007826218.1	-1.56	7.52E-05	hypothetical protein MAA_08220
19260542	MAA_06256	XM_007824254.1	-1.55	1.18E-02	proteasome-activating nucleotidase
19256624	MAA_02338	XM_007820336.1	-1.55	9.31E-07	General substrate transporter
19264597	MAA_10311	XM_007828309.1	-1.55	2.93E-03	Chloroperoxidase
19255658	MAA_01372	XM_007819370.1	-1.55	6.43E-05	hypothetical protein MBR_09810, partial
19254945	MAA_00659	XM_007818657.1	-1.54	2.61E-07	hypothetical protein MAA_00659
19257628	MAA_03342	XM_007821340.1	-1.53	2.32E-05	integral membrane protein
19254343	MAA_00057	XM_007818055.2	-1.53	1.21E-04	choline dehydrogenase
19261230	MAA_06944	XM_007824942.1	-1.53	1.00E-03	UDP-glucose 4-epimerase
19260855	MAA_06569	XM_007824567.2	-1.52	1.04E-03	AMP-dependent synthetase/ligase
19258281	MAA_03995	XM_007821993.1	-1.51	3.22E-03	ThiJ/PfpI family protein
19256453	MAA_02167	XM_007820165.2	-1.51	4.03E-02	Glucoamylase, putative
19264329	MAA_10043	XM_007828041.2	-1.49	3.54E-16	destruxin synthetase, DtxS1
19263759	MAA_09473	XM_007827471.2	-1.48	2.04E-06	DUF1338 domain protein
19255901	MAA_01615	XM_007819613.1	-1.48	2.09E-02	glucose oxidase
19257557	MAA_03271	XM_007821269.1	-1.47	2.24E-03	flavin-binding monooxygenase
19257582	MAA_03296	XM_007821294.1	-1.47	3.64E-10	hypothetical protein MAA_03296
19255066	MAA_00780	XM_007818778.1	-1.47	3.20E-05	zinc finger protein dhhc domain containing protein
19255553	MAA_01267	XM_007819265.1	-1.47	1.50E-03	hypothetical protein MAA_01267
19259755	MAA_05469	XM_007823467.1	-1.46	7.73E-06	siderophore iron transporter
19263128	MAA_08842	XM_007826840.1	-1.45	2.40E-03	NAD(P)-binding domain protein
19254740	MAA_00454	XM_007818452.1	-1.44	3.00E-04	Phospholipase C, phosphatidylinositol-specific, X domain protein
23632978	MAA_11530	XM_011413525.1	-1.44	6.81E-05	hypothetical protein X797_011732
23632221	MAA_10772	XM_011412691.1	-1.44	3.40E-03	cyclin-dependent kinase
19256971	MAA_02685	XM_007820683.1	-1.44	9.32E-09	chitinase
19259713	MAA_05427	XM_007823425.2	-1.43	1.63E-03	LysM domain-containing protein
19258825	MAA_04539	XM_007822635.1	-1.41	1.30E-05	Zn(2)-Cys(6) zinc finger domain protein
19263514	MAA_09228	XM_007827226.1	-1.40	8.17E-03	lysozyme
19256950	MAA_02664	XM_007820662.2	-1.40	1.74E-07	actin filament organization protein App1-like protein
19255871	MAA_01585	XM_007819583.2	-1.40	3.25E-02	hypothetical protein MAA_01585
19260070	MAA_05784	XM_007823782.1	-1.39	1.30E-04	hypothetical protein MAA_05784
19254364	MAA_00078	XM_007818076.2	-1.39	3.94E-02	FAD dependent monooxygenase
19261013	MAA_06727	XM_007824725.1	-1.39	5.97E-10	duf614 domain containing protein
19264752	MAA_10466	XM_007828464.1	-1.39	3.55E-05	Trimeric LpxA-like protein
19256610	MAA_02324	XM_007820322.1	-1.38	1.01E-02	hypothetical protein MAA_02324
19257586	MAA_03300	XM_007821298.1	-1.37	9.95E-08	Tetratricopeptide repeat protein
19263367	MAA_09081	XM_007827079.1	-1.37	3.73E-03	Rad21/Rec8 like protein
19261981	MAA_07695	XM_007825693.1	-1.37	7.26E-04	mitochondrial chaperone BCS1
19259921	MAA_05635	XM_007823633.1	-1.37	3.66E-04	CsbD-like protein
19264010	MAA_09724	XM_007827722.1	-1.37	4.85E-04	ATPase, P-type, potassium/sodium efflux, fungal
19256461	MAA_02175	XM_007820173.2	-1.36	1.09E-04	immunoglobulin I-set domain-containing protein
23632740	MAA_11292	XM_011413272.1	-1.36	1.49E-03	hypothetical protein MAA_11292
19261227	MAA_06941	XM_007824939.1	-1.35	6.29E-03	extracellular serine-rich protein
19262660	MAA_08374	XM_007826372.2	-1.34	9.35E-03	ankyrin repeat protein
19261596	MAA_07310	XM_007825308.2	-1.34	5.44E-04	integral membrane family protein
19264499	MAA_10213	XM_007828211.1	-1.34	1.72E-02	Cytochrome P450 CYP5317A1
23632695	MAA_11247	XM_011413227.1	-1.34	2.10E-02	hypothetical protein MAA_11247
19261280	MAA_06994	XM_007824992.1	-1.33	6.14E-04	secretory lipase
19257069	MAA_02783	XM_007820781.1	-1.32	4.76E-05	mucoidy inhibitor A
19255482	MAA_01196	XM_007819194.1	-1.32	2.84E-08	cysteine-rich secreted protein
19262758	MAA_08472	XM_007826470.1	-1.32	1.04E-02	Glycerate kinase
19260050	MAA_05764	XM_007823762.1	-1.31	4.61E-04	ThiJ/PfpI

19262103	MAA_07817	XM_007825815.1	-1.31	4.09E-04	glycoside hydrolase family 76 protein
19260235	MAA_05949	XM_007823947.1	-1.31	3.63E-07	cation transporter
19259756	MAA_05470	XM_007823468.2	-1.30	3.09E-02	transferase family protein
19263703	MAA_09417	XM_007827415.1	-1.30	1.85E-06	oligopeptide transporter
19254593	MAA_00307	XM_007818305.2	-1.30	1.74E-04	siderophore iron transporter
19257345	MAA_03059	XM_007821057.2	-1.29	9.90E-03	meiotically up-regulated 65 protein
19262044	MAA_07758	XM_007825756.1	-1.29	2.85E-04	coagulation factor 5/8 type domain protein
19259139	MAA_04853	XM_007822949.1	-1.28	1.51E-03	hypothetical protein MAA_04853
19254294	MAA_00008	XM_007818006.1	-1.27	9.32E-03	Alpha-L-arabinofuranosidase B, catalytic
19254962	MAA_00676	XM_007818674.1	-1.27	6.26E-08	hypothetical protein MAA_00676
19261242	MAA_06956	XM_007824954.1	-1.26	7.23E-03	hypothetical protein MAA_06956
19256488	MAA_02202	XM_007820200.2	-1.26	1.95E-08	zinc finger protein 58
19259306	MAA_05020	XM_007822531.2	-1.25	2.25E-06	phospholipase-like protein
19259557	MAA_05271	XM_007823269.1	-1.24	1.03E-07	nicotinamide N-methyltransferase
19261083	MAA_06797	XM_007824795.1	-1.24	1.80E-07	peptidase family M28
19256497	MAA_02211	XM_007820209.1	-1.24	5.28E-04	hexokinase-1
19262418	MAA_08132	XM_007826130.1	-1.24	3.83E-03	hypothetical protein MAA_08132
19258721	MAA_04435	XM_007822433.1	-1.23	7.82E-04	Salicylate synthase
19258291	MAA_04005	XM_007822003.1	-1.23	6.33E-06	Endopolyphosphatase, Ppn1p-related protein
19257316	MAA_03030	XM_007821028.1	-1.22	5.83E-04	hypothetical protein MAA_03030
19264231	MAA_09945	XM_007827943.1	-1.22	2.95E-02	c6 zinc finger domain containing protein
19254739	MAA_00453	XM_007818451.1	-1.22	3.61E-04	Ribonuclease T2, active site protein
19260913	MAA_06627	XM_007824625.1	-1.22	4.96E-04	hypothetical protein MAA_06627
19256757	MAA_02471	XM_007820469.1	-1.22	1.18E-05	phosducin
19255336	MAA_01050	XM_007819048.1	-1.22	5.78E-05	hypothetical protein MAA_01050
19257739	MAA_03453	XM_007821451.2	-1.21	2.18E-05	fungal specific transcription factor
19263892	MAA_09606	XM_007827604.2	-1.21	1.05E-02	FAD binding domain-containing protein
19263922	MAA_09636	XM_007827634.1	-1.21	3.14E-04	subtilisin-like protease
19257115	MAA_02829	XM_007820827.2	-1.20	5.70E-09	pali-domain-containing protein
19255487	MAA_01201	XM_007819199.1	-1.20	1.16E-03	Ctr copper transporter
19263958	MAA_09672	XM_007827670.2	-1.20	7.73E-06	WD domain-containing protein
19258805	MAA_04519	XM_007822615.2	-1.19	6.82E-03	caspase domain-containing protein
19259548	MAA_05262	XM_007823260.1	-1.19	1.28E-04	Mitochondrial substrate/solute carrier
19255970	MAA_01684	XM_007819682.1	-1.19	2.99E-04	Leucine Rich Repeat domain protein
19263848	MAA_09562	XM_007827560.1	-1.18	2.15E-02	fatty acid hydroxylase superfamily protein
19261382	MAA_07096	XM_007825094.2	-1.18	1.29E-02	hypothetical protein MAA_07096
19255800	MAA_01514	XM_007819512.1	-1.18	5.15E-06	Peptidase A1
19257045	MAA_02759	XM_007820757.2	-1.18	3.29E-06	Ca2+ permeable channel
19258127	MAA_03841	XM_007821839.1	-1.18	1.22E-02	Cytochrome P450 CYP6003A1
19265003	MAA_10719	XM_011412437.1	-1.18	6.89E-07	YTH domain protein
19255236	MAA_00950	XM_007818948.1	-1.17	2.46E-08	hypothetical protein MAA_00950
19259920	MAA_05634	XM_007823632.1	-1.17	9.95E-04	Src like protein
19261351	MAA_07065	XM_007825063.2	-1.17	4.09E-02	hypothetical protein MAA_07065
19257265	MAA_02979	XM_007820977.1	-1.17	1.25E-06	hypothetical protein MANI_024329
19264076	MAA_09790	XM_007827788.1	-1.16	1.26E-02	hypothetical protein MAA_09790
19258535	MAA_04249	XM_007822247.2	-1.16	1.25E-02	methyltransferase
19258280	MAA_03994	XM_007821992.1	-1.15	2.65E-02	glycosyl transferase GT-A type structural fold protein
19258514	MAA_04228	XM_007822226.1	-1.15	1.37E-05	phospho-2-dehydro-3-deoxyheptonate aldolase
19262192	MAA_07906	XM_007825904.1	-1.15	7.18E-03	Alpha-hydroxy acid dehydrogenase, FMN-dependent
23632203	MAA_10754	XM_011412472.1	-1.15	3.66E-02	gag-pol polyprotein
19257842	MAA_03556	XM_007821554.1	-1.15	5.85E-04	hypothetical protein H634G_07741
19255594	MAA_01308	XM_007819306.2	-1.14	8.18E-06	Peptidase S8/S53, subtilisin/kexin/sedolisin
19259541	MAA_05255	XM_007823253.2	-1.14	4.18E-02	isopenicillin N synthase-like protein
19261293	MAA_07007	XM_007825005.2	-1.14	3.89E-04	alkaline phosphatase-like protein
19255473	MAA_01187	XM_007819185.1	-1.13	3.78E-06	DUF2205 domain protein
19257577	MAA_03291	XM_007821289.1	-1.13	7.12E-04	Zn(2)-C6 fungal-type DNA-binding domain protein
19263485	MAA_09199	XM_007827197.1	-1.12	4.92E-04	ATP-dependent RNA helicase DED1
19264476	MAA_10190	XM_007828188.1	-1.12	4.60E-03	Heterokaryon incompatibility
19263310	MAA_09024	XM_007827022.1	-1.11	3.77E-03	selenium-binding protein
23632752	MAA_11304	XM_011413292.1	-1.11	4.03E-02	maleylacetate reductase
19260910	MAA_06624	XM_007824622.2	-1.11	1.85E-02	lysophospholipase precursor

19254358	MAA_00072	XM_007818070.1	-1.10	2.40E-03	hypothetical protein MAA_00072
19254417	MAA_00131	XM_007818129.1	-1.10	3.31E-04	hypothetical protein MAA_00131
19263684	MAA_09398	XM_007827396.1	-1.10	5.56E-03	Ankyrin repeat protein
19261473	MAA_07187	XM_007825185.1	-1.09	1.24E-11	expression library immunization antigen 1
19262532	MAA_08246	XM_007826244.1	-1.09	1.42E-04	ankyrin repeat domain containing protein
19262380	MAA_08094	XM_007826092.1	-1.09	1.57E-04	hypothetical protein MAA_08094
19264416	MAA_10130	XM_007828128.2	-1.09	1.29E-02	Major facilitator superfamily domain, general substrate transporter
19260886	MAA_06600	XM_007824598.2	-1.09	1.68E-02	hypothetical protein MAA_06600
19254534	MAA_00248	XM_007818246.1	-1.09	1.17E-02	HMG (high mobility group) box protein
19262271	MAA_07985	XM_007825983.1	-1.09	8.81E-03	carboxylesterase/lipase domain protein
19259102	MAA_04816	XM_007822912.1	-1.08	1.97E-07	cation transporting ATPase
19257500	MAA_03214	XM_007821212.1	-1.08	2.47E-05	Amino acid transporter, transmembrane
19263655	MAA_09369	XM_007827367.1	-1.08	4.74E-02	hypothetical protein MAA_09369
19263915	MAA_09629	XM_007827627.1	-1.08	1.42E-06	Glycoside hydrolase, family 35
19261772	MAA_07486	XM_007825484.2	-1.07	3.66E-02	CFEM domain protein
19261097	MAA_06811	XM_007824809.1	-1.07	1.11E-03	hypothetical protein H634G_06914
19255698	MAA_01412	XM_007819410.2	-1.06	5.14E-04	Ankyrin repeat-containing domain protein
19255138	MAA_00852	XM_007818850.2	-1.06	1.30E-02	swim zinc finger domain protein
19256697	MAA_02411	XM_007820409.2	-1.06	8.20E-03	alpha glucoside transporter
19260351	MAA_06065	XM_007824063.1	-1.06	2.47E-02	Basic-leucine zipper (bZIP) transcription factor
19260432	MAA_06146	XM_007824144.2	-1.06	6.92E-10	glycoside hydrolase family 3 protein
19256409	MAA_02123	XM_007820121.1	-1.06	6.71E-09	WW domain-binding protein
19263194	MAA_08908	XM_007826906.1	-1.06	2.85E-04	FHA domain containing protein
19260803	MAA_06517	XM_007824515.1	-1.05	1.43E-02	protein pyrABCN
19261231	MAA_06945	XM_007824943.2	-1.05	1.23E-03	glycosyltransferase family 4 protein
19256410	MAA_02124	XM_007820122.1	-1.05	1.56E-09	menaquinone methyltransferase related to dlpA protein
19260619	MAA_06333	XM_007824331.2	-1.05	1.43E-02	AAA family ATPase
19264348	MAA_10062	XM_007828060.1	-1.05	6.05E-03	polyprotein
19256077	MAA_01791	XM_007819789.1	-1.05	2.89E-02	Major facilitator superfamily domain protein
19262933	MAA_08647	XM_007826645.2	-1.04	4.51E-02	hypothetical protein MAA_08647
19261509	MAA_07223	XM_007825221.1	-1.04	6.51E-04	hypothetical protein MAA_07223
23632275	MAA_10826	XM_011412745.1	-1.04	9.24E-03	meiotic recombination protein DMC1
19260739	MAA_06453	XM_007824451.1	-1.04	2.93E-03	GCN5-related N-acetyltransferase (GNAT) domain protein
19262238	MAA_07952	XM_007825950.1	-1.03	2.26E-02	Glycosyl transferase, family 25
19259442	MAA_05156	XM_007823154.1	-1.03	9.85E-04	hypothetical protein H634G_04326
19262454	MAA_08168	XM_007826166.2	-1.03	8.43E-03	Peptidase S8, subtilisin-related protein
23632270	MAA_10821	XM_011412740.1	-1.03	5.64E-03	ubiquitin-conjugating enzyme
19262129	MAA_07843	XM_007825841.1	-1.02	6.49E-03	patatin-like phospholipase
23632739	MAA_11291	XM_011413271.1	-1.02	6.47E-03	hypothetical protein MAA_11291
19258332	MAA_04046	XM_007822044.1	-1.02	1.34E-02	RNA recognition motif domain protein
19256214	MAA_01928	XM_007819926.1	-1.02	4.42E-02	Ubiquitin 3 binding protein But2
19255636	MAA_01350	XM_007819348.2	-1.02	1.05E-02	general substrate transporter
19255412	MAA_01126	XM_007819124.1	-1.02	4.99E-04	Alpha/beta hydrolase fold-3
19255071	MAA_00785	XM_007818783.1	-1.02	1.29E-04	annexin ANXC4
19264538	MAA_10252	XM_007828250.1	-1.02	3.72E-04	magnesium-translocating P-type ATPase
19259926	MAA_05640	XM_007823638.1	-1.01	1.03E-02	aldo-keto reductase family protein
19261480	MAA_07194	XM_007825192.1	-1.01	6.88E-06	hypothetical protein MAA_07194
19263306	MAA_09020	XM_007827018.1	-1.01	1.16E-02	hypothetical protein MAA_09020
19258202	MAA_03916	XM_007821914.2	-1.00	4.09E-02	proliferating cell nuclear antigen
19258435	MAA_04149	XM_007822147.1	-1.00	2.25E-03	Armadillo-type fold protein
19260940	MAA_06654	XM_007824652.1	-1.00	2.57E-11	ferrioxamine B transporter
19264829	MAA_10543	XM_007828541.1	1.00	4.85E-13	aminoglycoside 3'-phosphotransferase/choline kinase domain protein
19261806	MAA_07520	XM_007825518.2	1.00	2.23E-03	major facilitator superfamily transporter
23632363	MAA_10914	XM_011412860.1	1.00	1.58E-02	pol-like protein
19263400	MAA_09114	XM_007827112.2	1.01	5.60E-05	Ribonuclease H-like protein
19255584	MAA_01298	XM_007819296.1	1.01	1.81E-02	O-methyltransferase
19262654	MAA_08368	XM_007826366.2	1.01	1.85E-04	major facilitator superfamily transporter
19264801	MAA_10515	XM_007828513.1	1.01	4.57E-04	Metallopeptidase, catalytic domain protein
23633182	MAA_11734	XM_011412649.1	1.01	2.00E-08	hypothetical protein MAA_11734
19264830	MAA_10544	XM_007828542.1	1.01	6.37E-12	Protein kinase, ATP binding site
19261146	MAA_06860	XM_007824858.2	1.01	1.11E-03	F-box domain protein

19263397	MAA_09111	XM_007827109.1	1.01	1.87E-04	NUDIX hydrolase domain protein
19258353	MAA_04067	XM_007822065.1	1.02	1.51E-06	dipeptidyl peptidase
19263535	MAA_09249	XM_007827247.1	1.02	5.86E-03	beta-lactamase
19260841	MAA_06555	XM_007824553.1	1.02	2.03E-03	methyltransferase
23633238	MAA_11790	XM_011412978.1	1.02	1.80E-03	Zn(2)-Cys(6) zinc finger domain protein, partial
19264018	MAA_09732	XM_007827730.1	1.02	4.44E-03	Aldo/keto reductase
19263316	MAA_09030	XM_007827028.1	1.02	1.81E-02	Sodium/solute symporter
19263890	MAA_09604	XM_007827602.1	1.02	1.02E-03	Ig domain protein group 2 domain protein
19263221	MAA_08935	XM_007826933.1	1.04	5.88E-04	Killer toxin, Kp4/SMK-like, core
19264907	MAA_10621	XM_011412339.1	1.04	1.64E-02	MYB DNA-binding domain-containing protein
23632362	MAA_10913	XM_011412859.1	1.04	2.43E-08	hypothetical protein MAA_10913
19263417	MAA_09131	XM_007827129.1	1.04	7.14E-06	zinc finger domain-containing protein
19262889	MAA_08603	XM_007826601.2	1.04	4.53E-06	transcription factor Cys6
19264584	MAA_10298	XM_007828296.1	1.04	2.25E-02	hydrophobin
23633286	MAA_11838	XM_011413366.1	1.04	5.78E-05	Ankyrin repeat-containing domain protein
23633159	MAA_11711	XM_011412626.1	1.04	1.09E-03	zinc finger domain-containing protein
19263978	MAA_09692	XM_007827690.1	1.04	1.29E-03	Oxoglutarate/iron-dependent oxygenase
19256915	MAA_02629	XM_007820627.1	1.05	6.12E-03	Alcohol dehydrogenase superfamily, zinc-type
19263391	MAA_09105	XM_007827103.2	1.06	2.11E-07	hypothetical protein MAA_09105
19262673	MAA_08387	XM_007826385.1	1.06	8.09E-04	short-chain dehydrogenase
19262471	MAA_08185	XM_007826183.1	1.06	5.37E-04	LEA domain protein
19255186	MAA_00900	XM_007818898.1	1.06	5.32E-04	hypothetical protein MAA_00900
19255677	MAA_01391	XM_007819389.1	1.06	1.23E-04	transposase
23632365	MAA_10916	XM_011412862.1	1.06	1.38E-02	Ribonuclease H-like protein
23633205	MAA_11757	XM_011412672.1	1.07	3.14E-04	hypothetical protein MAA_11757
19259242	MAA_04956	XM_007822467.1	1.07	2.14E-05	Amine oxidase
19255392	MAA_01106	XM_007819104.2	1.07	1.30E-08	Ubiquitin supergroup
19262964	MAA_08678	XM_007826676.1	1.07	2.87E-02	hypothetical protein MAA_08678
23632906	MAA_11458	XM_011413452.1	1.07	4.41E-06	hypothetical protein MAA_11458
19258249	MAA_03963	XM_007821961.1	1.08	1.16E-03	hypothetical protein MAA_03963
23633141	MAA_11693	XM_011412608.1	1.08	9.84E-06	hypothetical protein MAA_11693
23632274	MAA_10825	XM_011412744.1	1.08	2.85E-06	hypothetical protein MAA_10825
23633175	MAA_11727	XM_011412642.1	1.08	6.20E-15	FluG domain-containing protein
19257788	MAA_03502	XM_007821500.1	1.09	3.58E-03	zeta toxin family protein
19262692	MAA_08406	XM_007826404.1	1.10	1.22E-03	glycoside hydrolase family 16
19263390	MAA_09104	XM_007827102.2	1.10	1.93E-10	aaa ATPase
23633188	MAA_11740	XM_011412655.1	1.10	3.74E-14	Zn(2)-C6 fungal-type DNA-binding domain protein
19255688	MAA_01402	XM_007819400.1	1.10	5.35E-07	hypothetical protein MAA_01402
23633179	MAA_11731	XM_011412646.1	1.11	2.50E-15	hypothetical protein MAA_11731
23633142	MAA_11694	XM_011412609.1	1.11	9.35E-16	hypothetical protein MAA_11694
19256318	MAA_02032	XM_007820030.2	1.11	8.43E-03	3-phytase
19255772	MAA_01486	XM_007819484.1	1.11	4.18E-04	serine carboxypeptidase
19254667	MAA_00381	XM_007818379.2	1.11	1.81E-02	Ribonuclease H-like protein
19256665	MAA_02379	XM_007820377.2	1.12	2.77E-03	Aromatic-ring hydroxylase-like protein
19263423	MAA_09137	XM_007827135.1	1.12	1.03E-13	Protein kinase-like protein
19264721	MAA_10435	XM_007828433.2	1.12	9.32E-05	sulfite oxidase
19259511	MAA_05225	XM_007823223.2	1.12	1.74E-03	extracellular dipeptidyl-peptidase Dpp4
19259248	MAA_04962	XM_007822473.1	1.12	3.00E-04	aldehyde dehydrogenase
19263392	MAA_09106	XM_007827104.1	1.12	4.82E-14	FluG domain-containing protein
19258466	MAA_04180	XM_007822178.2	1.12	1.50E-02	TfdA family Taurine catabolism dioxygenase TauD
19264293	MAA_10007	XM_007828005.1	1.13	9.03E-04	WD40 repeat-like-containing domain protein
19263219	MAA_08933	XM_007826931.1	1.13	8.38E-04	Killer toxin, Kp4/SMK-like, core
19264434	MAA_10148	XM_007828146.2	1.13	4.59E-03	ATP-dependent DNA helicase PIF1
19262824	MAA_08538	XM_007826536.2	1.13	1.12E-02	3-hydroxyacid dehydrogenase/reductase
19258184	MAA_03898	XM_007821896.2	1.14	1.71E-02	long-chain-fatty-acid-CoA ligase
23632224	MAA_10775	XM_011412694.1	1.14	3.04E-02	hypothetical protein MAA_10775
19255889	MAA_01603	XM_007819601.2	1.14	9.06E-10	hypothetical protein MAA_01603
19264450	MAA_10164	XM_007828162.2	1.14	7.77E-09	reverse transcriptase, RNaseH
19263386	MAA_09100	XM_007827098.2	1.14	4.04E-07	DDE superfamily endonuclease, CENP-B-like protein
19257778	MAA_03492	XM_007821490.2	1.14	2.03E-05	feruloyl esterase B
19262472	MAA_08186	XM_007826184.1	1.14	4.55E-02	late embryogenesis abundant protein

19258564	MAA_04278	XM_007822276.1	1.15	2.04E-02	hypothetical protein MAA_04278
19264062	MAA_09776	XM_007827774.1	1.15	1.46E-05	lysine amidinotransferase
19254683	MAA_00397	XM_007818395.1	1.15	2.17E-03	Protein kinase-like protein
19264024	MAA_09738	XM_007827736.1	1.15	1.04E-05	ThiJ/PfpI family protein
19263888	MAA_09602	XM_007827600.1	1.16	3.44E-03	Six-hairpin glycosidase
19263266	MAA_08980	XM_007826978.1	1.16	3.06E-03	Cyanovirin-N
19255217	MAA_00931	XM_007818929.1	1.16	5.94E-12	Ribonuclease H-like protein
19263403	MAA_09117	XM_007827115.1	1.17	5.37E-10	hypothetical protein MAA_09117
19263692	MAA_09406	XM_007827404.1	1.17	2.94E-06	hypothetical protein MAA_09406
19263977	MAA_09691	XM_007827689.1	1.17	1.15E-03	1-aminocyclopropane-1-carboxylate synthase
19264826	MAA_10540	XM_007828538.1	1.17	5.62E-03	hypothetical protein MAA_10540
19263358	MAA_09072	XM_007827070.1	1.17	1.39E-02	cell wall galactomannoprotein Mp2/allergen F17-like protein
23633132	MAA_11684	XM_011412599.1	1.18	1.82E-07	hypothetical protein MAA_11684
19260840	MAA_06554	XM_007824552.2	1.19	2.58E-13	delta-12 fatty acid desaturase
19260584	MAA_06298	XM_007824296.2	1.19	1.72E-04	MFS transporter [Metarhizium robertsii]
19263497	MAA_09211	XM_007827209.1	1.19	1.75E-04	hypothetical protein MAA_09211
19257789	MAA_03503	XM_007821501.2	1.19	4.93E-03	Protein kinase-like protein
23633172	MAA_11724	XM_011412639.1	1.20	4.76E-04	Short-chain dehydrogenase/reductase SDR
19264446	MAA_10160	XM_007828158.2	1.20	8.86E-16	transposase-like protein
19264772	MAA_10486	XM_007828484.1	1.20	2.14E-02	gamma-glutamyltranspeptidase periplasmic precursor
19260689	MAA_06403	XM_007824401.1	1.21	9.41E-06	glycoside hydrolase family 76 protein
19256359	MAA_02073	XM_007820071.1	1.21	6.62E-07	nuclear membrane fusion protein Kar5
19263164	MAA_08878	XM_007826876.2	1.21	1.29E-05	hypothetical protein MAA_08878
19255177	MAA_00891	XM_007818889.1	1.21	5.50E-03	hypothetical protein X797_011528
19264141	MAA_09855	XM_007827853.1	1.22	8.66E-04	4-hydroxyphenylpyruvate dioxygenase
23632510	MAA_11062	XM_011413028.1	1.22	2.47E-02	secretory phospholipase A2
19258471	MAA_04185	XM_007822183.1	1.22	2.60E-03	G-protein coupled receptor
19259142	MAA_04856	XM_007822952.1	1.23	4.47E-03	DUF323 domain protein
19259110	MAA_04824	XM_007822920.2	1.23	8.58E-09	non-ribosomal peptide synthetase
19263396	MAA_09110	XM_007827108.1	1.23	3.40E-11	uracil phosphoribosyltransferase
19264834	MAA_10548	XM_007828546.1	1.23	3.44E-04	ATP-dependent DNA helicase PIF1
19264818	MAA_10532	XM_007828530.1	1.23	2.06E-02	peptidase family M3
19263320	MAA_09034	XM_007827032.1	1.24	4.66E-03	L-xylulose reductase
19264445	MAA_10159	XM_007828157.1	1.24	5.66E-08	restless-like transposase
19255187	MAA_00901	XM_007818899.2	1.24	1.44E-09	hypothetical protein MAA_00901
19259838	MAA_05552	XM_007823550.1	1.24	7.29E-03	ribosomal protein L1
19257096	MAA_02810	XM_007820808.2	1.24	2.14E-03	Fungal chitosanase
19260842	MAA_06556	XM_007824554.1	1.25	2.01E-12	Glutathione S-transferase
23633173	MAA_11725	XM_011412640.1	1.25	5.45E-04	Metallopeptidase, catalytic domain protein
19264954	MAA_10670	XM_011412388.1	1.26	2.10E-02	hypothetical protein MAA_10670
19264758	MAA_10472	XM_007828470.1	1.26	3.11E-09	gag-pol polyprotein
19261561	MAA_07275	XM_007825273.1	1.26	5.53E-10	hypothetical protein X797_006137
23632299	MAA_10850	XM_011412769.1	1.27	2.33E-05	hypothetical protein MAA_10850
19255997	MAA_01711	XM_007819709.2	1.27	8.68E-03	Beta-lactamase-like protein
19258830	MAA_04544	XM_007822640.2	1.27	2.15E-02	hypothetical protein MAA_04544
23633252	MAA_11804	XM_011412992.1	1.28	1.29E-02	hypothetical protein X797_012022
19264635	MAA_10349	XM_007828347.1	1.28	6.35E-16	ATP-dependent DNA helicase PIF1
19254778	MAA_00492	XM_007818490.1	1.28	1.05E-11	subtilisin-like protease
19263218	MAA_08932	XM_007826930.1	1.29	2.56E-05	hypothetical protein MAA_08932
19260869	MAA_06583	XM_007824581.2	1.29	5.66E-08	feruloyl esterase B
19257077	MAA_02791	XM_007820789.1	1.29	1.75E-07	Glutathione S-transferase/chloride channel
23633184	MAA_11736	XM_011412651.1	1.30	9.22E-04	patatin-like phospholipase
23632921	MAA_11473	XM_011413467.1	1.30	2.14E-03	Ankyrin repeat-containing domain protein
19259936	MAA_05650	XM_007823648.1	1.30	3.82E-02	MFS transporter
19263628	MAA_09342	XM_007827340.1	1.31	4.42E-02	carbohydrate-binding module family 13 protein
19263169	MAA_08883	XM_007826881.1	1.31	1.21E-02	FAD binding domain-containing protein
19262240	MAA_07954	XM_007825952.1	1.31	6.86E-05	hypothetical protein MAA_07954
19264577	MAA_10291	XM_007828289.2	1.32	7.14E-06	cyclopentanone 1,2-monooxygenase
23633241	MAA_11793	XM_011412981.1	1.33	5.83E-04	hypothetical protein MAA_11793
19261469	MAA_07183	XM_007825181.1	1.34	4.51E-03	hypothetical protein MAA_07183
19264070	MAA_09784	XM_007827782.1	1.34	3.99E-10	16 kDa allergen

19261690	MAA_07404	XM_007825402.1	1.35	1.72E-02	B-(1-6) glucan synthase
19254648	MAA_00362	XM_007818360.2	1.35	2.15E-07	PIF1 protein
19254780	MAA_00494	XM_007818492.1	1.36	2.93E-03	protein-arginine deiminase type-4
23633180	MAA_11732	XM_011412647.1	1.36	7.89E-05	hypothetical protein MAA_11732
19261170	MAA_06884	XM_007824882.1	1.36	4.58E-07	Aldehyde dehydrogenase
23632218	MAA_10769	XM_011412688.1	1.37	9.70E-03	Thioredoxin
19255583	MAA_01297	XM_007819295.1	1.38	2.87E-03	haloacid dehalogenase
19254384	MAA_00098	XM_007818096.1	1.39	1.62E-02	FluG domain-containing protein
19260837	MAA_06551	XM_007824549.1	1.39	1.79E-13	macrophomate synthase
23632196	MAA_10747	XM_011412465.1	1.39	3.28E-03	ankyrin repeat protein
19258326	MAA_04040	XM_007822038.1	1.41	3.83E-03	alpha/beta hydrolase
19256358	MAA_02072	XM_007820070.1	1.41	3.01E-04	Cytochrome P450
23632364	MAA_10915	XM_011412861.1	1.41	1.01E-15	hypothetical protein MAA_10915
23633185	MAA_11737	XM_011412652.1	1.42	2.34E-02	hypothetical protein MAA_11737
19264853	MAA_10567	XM_007828565.1	1.42	3.77E-04	restless-like transposase
19263023	MAA_08737	XM_007826735.1	1.42	3.28E-05	Beta-lactamase-like protein
19264442	MAA_10156	XM_007828154.1	1.43	9.69E-15	hypothetical protein MAA_10156
19254392	MAA_00106	XM_007818104.1	1.44	3.88E-03	F-box domain protein
19264325	MAA_10039	XM_007828037.1	1.44	2.43E-04	subgroup IIIi aminotransferase
19263202	MAA_08916	XM_007826914.2	1.45	4.02E-04	multicopper oxidase
19254406	MAA_00120	XM_007818118.2	1.45	7.43E-18	Methyltransferase type 11
19264066	MAA_09780	XM_007827778.1	1.45	3.92E-02	glycosyl transferase, group 2 family protein
19262832	MAA_08546	XM_007826544.1	1.46	8.20E-03	Amidase
19260260	MAA_05974	XM_007823972.1	1.46	4.72E-04	S-adenosylmethionine:tRNA ribosyltransferase-isomerase
19262653	MAA_08367	XM_007826365.2	1.47	4.92E-04	ERG4/ERG24 ergosterol biosynthesis-like protein
19264827	MAA_10541	XM_007828539.1	1.47	2.39E-14	hypothetical protein MAA_10541
19263793	MAA_09507	XM_007827505.1	1.48	8.24E-03	umta
23632323	MAA_10874	XM_011412793.1	1.48	3.49E-05	hypothetical protein MAA_10874
19263398	MAA_09112	XM_007827110.1	1.48	5.82E-21	hypothetical protein MAA_09112
23632495	MAA_11047	XM_011413013.1	1.49	1.42E-17	hypothetical protein MAA_11047
19263587	MAA_09301	XM_007827299.2	1.49	3.35E-02	F-box domain-containing protein
19264448	MAA_10162	XM_007828160.2	1.50	1.25E-10	transposase-like protein
19262651	MAA_08365	XM_007826363.1	1.51	2.02E-05	multicopper oxidase
19258027	MAA_03741	XM_007821739.2	1.57	1.62E-02	Major facilitator superfamily domain, general substrate transporter
19258327	MAA_04041	XM_007822039.2	1.61	1.26E-04	hypothetical protein MAA_04041
19256546	MAA_02260	XM_007820258.1	1.62	1.28E-07	sarcosine oxidase
19260261	MAA_05975	XM_007823973.1	1.62	5.50E-03	(2R)-phospho-3-sulfolactate synthase, ComA
19265002	MAA_10718	XM_011412436.1	1.64	3.24E-06	hypothetical protein MAA_10718
19263904	MAA_09618	XM_007827616.1	1.65	1.89E-04	CobW domain protein
19255201	MAA_00915	XM_007818913.1	1.66	6.20E-06	Ribonuclease H-like protein
19262822	MAA_08536	XM_007826534.2	1.68	1.23E-05	multidrug resistance protein 1
19264611	MAA_10325	XM_007828323.1	1.69	1.80E-03	Nitrilase/cyanide hydratase and apolipoprotein N-acyltransferase
19255639	MAA_01353	XM_007819351.1	1.69	1.41E-05	Oxoglutarate/iron-dependent oxygenase
19262963	MAA_08677	XM_007826675.2	1.69	1.63E-02	hypothetical protein MAA_08677
23633181	MAA_11733	XM_011412648.1	1.69	1.25E-10	GTP cyclohydrolase family protein
19254647	MAA_00361	XM_007818359.1	1.71	7.92E-15	transposase-like protein
19255666	MAA_01380	XM_007819378.1	1.71	2.14E-02	uncharacterized protein MAA_01380
23633070	MAA_11622	XM_011412537.1	1.71	7.07E-04	hypothetical protein MAA_11622
19264142	MAA_09856	XM_007827854.1	1.72	4.94E-05	hypothetical protein MAA_09856
19264386	MAA_10100	XM_007828098.1	1.72	1.92E-02	hypothetical protein MAA_10100
19262890	MAA_08604	XM_007826602.2	1.74	1.51E-11	hypothetical protein MAA_08604
19258065	MAA_03779	XM_007821777.2	1.79	4.18E-10	peptidylarginine deiminase-like enzyme
19263659	MAA_09373	XM_007827371.1	1.79	6.55E-03	hypothetical protein MAA_09373
19262652	MAA_08366	XM_007826364.1	1.80	3.44E-07	methyltransferase
19262904	MAA_08618	XM_007826616.1	1.81	1.57E-02	Phytanoyl-CoA dioxygenase PhyH
19255194	MAA_00908	XM_007818906.1	1.81	4.99E-03	might be a transmembrane protein
19263545	MAA_09259	XM_007827257.1	1.84	2.37E-06	epoxidase subunit A
19262906	MAA_08620	XM_007826618.1	1.84	2.49E-02	nmrA-like family protein
19255954	MAA_01668	XM_007819666.2	1.84	6.33E-05	Phosphoesterase
19255690	MAA_01404	XM_007819402.1	1.84	5.48E-36	hypothetical protein MAA_01404
19254771	MAA_00485	XM_007818483.1	1.86	3.91E-02	hypothetical protein MAA_00485

19262903	MAA_08617	XM_007826615.2	1.87	3.28E-02	aromatic amino acid aminotransferase
19260829	MAA_06543	XM_007824541.2	1.87	8.95E-10	cytochrome P450 [Metarhizium robertsii]
19263874	MAA_09588	XM_007827586.2	1.89	1.91E-05	TfdA family Taurine catabolism dioxygenase TauD
19263645	MAA_09359	XM_007827357.2	1.89	2.82E-04	Pfs, NACHT and WD domain protein
19261010	MAA_06724	XM_007824722.1	1.90	2.55E-05	RmlC-like jelly roll fold protein
19264734	MAA_10448	XM_007828446.1	1.92	1.87E-05	hydrolase
19260313	MAA_06027	XM_007824025.1	1.92	7.88E-17	type 11 methyltransferase
19263747	MAA_09461	XM_007827459.1	1.93	9.96E-19	carbohydrate-binding WSC domain protein
23633210	MAA_11762	XM_011412677.1	1.93	5.16E-04	reverse transcriptase
19257622	MAA_03336	XM_007821334.1	1.94	2.40E-07	Peptidase S8/S53, subtilisin/kexin/sedolisin
19255582	MAA_01296	XM_007819294.1	1.94	2.70E-02	Cytochrome P450 CYP620N1
19262577	MAA_08291	XM_007826289.2	1.94	2.49E-04	sulfatase domain-containing protein
19264817	MAA_10531	XM_007828529.1	1.95	1.62E-05	Oxoglutarate/iron-dependent oxygenase
19258293	MAA_04007	XM_007822005.2	1.95	2.27E-08	alcohol dehydrogenase GroES domain-containing protein
19262927	MAA_08641	XM_007826639.2	1.97	4.02E-03	sulfatase domain protein
19262905	MAA_08619	XM_007826617.2	1.97	5.09E-07	choline transport protein
19261978	MAA_07692	XM_007825690.1	1.97	1.30E-04	carbohydrate-binding protein, partial
19264603	MAA_10317	XM_007828315.1	1.98	7.41E-10	pyridoxal-dependent decarboxylase domain protein
19263643	MAA_09357	XM_007827355.2	1.99	3.54E-02	hypothetical protein X797_010135
19262974	MAA_08688	XM_007826686.1	2.00	1.94E-11	CFEM domain protein
19261356	MAA_07070	XM_007825068.1	2.01	9.06E-23	hypothetical protein MAA_07070
19256607	MAA_02321	XM_007820319.1	2.02	8.58E-12	Major facilitator superfamily domain, general substrate transporter
19262655	MAA_08369	XM_007826367.1	2.07	9.14E-09	Dimeric alpha-beta barrel
19264481	MAA_10195	XM_007828193.2	2.07	1.54E-04	Ctr copper transporter
19260312	MAA_06026	XM_007824024.1	2.08	4.42E-12	phosphatidylserine decarboxylase family protein
19264464	MAA_10178	XM_007828176.1	2.10	1.30E-08	Cyclin PHO80-like protein
23632628	MAA_11180	XM_011413146.1	2.13	1.14E-03	Cellobiose dehydrogenase, cytochrome
19262559	MAA_08273	XM_007826271.1	2.16	1.34E-08	PA14 domain protein
19262218	MAA_07932	XM_007825930.1	2.17	4.74E-03	Pyruvate/2-oxoglutarate dehydrogenase
19262909	MAA_08623	XM_007826621.1	2.21	4.72E-02	phytanoyl-CoA dioxygenase
23632994	MAA_11546	XM_011413541.1	2.22	1.69E-07	hypothetical protein MAA_11546
19264319	MAA_10033	XM_007828031.2	2.28	1.83E-10	polyketide synthase
19260992	MAA_06706	XM_007824704.1	2.30	3.66E-03	short-chain dehydrogenase
19262907	MAA_08621	XM_007826619.1	2.31	1.22E-02	f420-dependent NADP reductase
19258452	MAA_04166	XM_007822164.1	2.35	3.94E-08	DUF1275 domain protein
19255985	MAA_01699	XM_007819697.1	2.40	1.03E-07	serin endopeptidase
19264505	MAA_10219	XM_007828217.2	2.44	2.22E-02	FAD/FMN-containing isoamyl alcohol oxidase MreA
19260311	MAA_06025	XM_007824023.2	2.51	1.02E-12	PRO1A C6 Zink-finger protein
23633189	MAA_11741	XM_011412656.1	2.75	3.55E-36	hypothetical protein MAA_11741
19264326	MAA_10040	XM_007828038.1	2.84	2.30E-11	Zn(2)-Cys(6) zinc finger domain protein
19258063	MAA_03777	XM_007821775.1	2.87	4.00E-02	Bys1 family protein
19262560	MAA_08274	XM_007826272.2	2.91	1.13E-12	hypothetical protein MAA_08274
19256813	MAA_02527	XM_007820525.1	2.96	1.06E-03	hypothetical protein MAA_02527
19264126	MAA_09840	XM_007827838.1	3.01	3.69E-02	hypothetical protein MAA_09840
23633270	MAA_11822	XM_011413159.1	3.16	1.86E-12	GPCR, PTH-type
19255481	MAA_01195	XM_007819193.1	3.17	2.62E-07	cytosine deaminase
19255706	MAA_01420	XM_007819418.1	3.28	2.27E-12	hypothetical protein MAA_01420
19255149	MAA_00863	XM_007818861.2	3.53	1.78E-03	Protein kinase-like protein
19264610	MAA_10324	XM_007828322.1	3.62	3.70E-03	NmrA-like family protein
19264812	MAA_10526	XM_007828524.1	3.71	4.03E-02	hypothetical protein MAA_10526
19261429	MAA_07143	XM_007825141.1	3.99	2.93E-02	DUF946 domain-containing protein
19255705	MAA_01419	XM_007819417.1	4.01	2.64E-03	Calcium-mediated lectin
19261893	MAA_07607	XM_007825605.1	4.56	2.71E-24	hypothetical protein MAA_07607
19257555	MAA_03269	XM_007821267.2	5.05	3.68E-02	membrane transporter
19255348	MAA_01062	XM_007819060.2	7.81	2.89E-37	hypothetical protein MAA_01062
19264403	MAA_10117	XM_007828115.1	10.70	1.17E-02	Zn(2)-C6 fungal-type DNA-binding domain protein
19262821	MAA_08535	XM_007826533.1	11.37	2.47E-02	tripeptidyl peptidase a
19264488	MAA_10202	XM_007828200.2	14.39	4.08E-10	Beta-ketoacyl synthase

Table S3. GO analysis of differentially expressed genes in *M. robertsii* Δ*cfp1* versus WT strain.

GO_ID	GO_class	*p*-value	Gene_ID	Gene_name	$\log_2 R$	Annotation
GO:0005575	2	6.61E-06	19262961	MAA_08675	-14.89	hypothetical protein MAA_08675
GO:0005575	2	6.61E-06	19261241	MAA_06955	-11.71	lipase 2
GO:0005575	2	6.61E-06	19262574	MAA_08288	-11.53	Cyclin-like F-box
GO:0005575	2	6.61E-06	19254642	MAA_00356	-10.67	glycosyl transferase
GO:0005575	2	6.61E-06	19264862	MAA_10576	-10.62	Uncharacterized protein MAA_10576
GO:0005575	2	6.61E-06	19255828	MAA_01542	-10.54	hypothetical protein MAA_01542
GO:0005575	2	6.61E-06	19262985	MAA_08699	-8.86	Beta-ketoacyl synthase
GO:0005575	2	6.61E-06	19261770	MAA_07484	-7.95	peptidase alpha-lytic pro domain protein
GO:0005575	2	6.61E-06	19262600	MAA_08314	-5.35	ATPase
GO:0005575	2	6.61E-06	19259182	MAA_04896	-5.06	regulatory P domain-containing protein
GO:0005575	2	6.61E-06	19260861	MAA_06575	-4.95	Beta-ketoacyl synthase
GO:0005575	2	6.61E-06	19255257	MAA_00971	-4.81	Aromatic prenyltransferase, DMATS type
GO:0005575	2	6.61E-06	19264560	MAA_10274	-4.58	TAM domain methyltransferase
GO:0005575	2	6.61E-06	19262115	MAA_07829	-4.38	WD40 repeat-like-containing domain protein
GO:0005575	2	6.61E-06	23633214	MAA_11766	-3.86	hypothetical protein MAA_11766
GO:0005575	2	6.61E-06	19257526	MAA_03240	-3.79	hypothetical protein MAA_03240
GO:0005575	2	6.61E-06	19264332	MAA_10046	-3.68	Aspartic acid decarboxylase, DtxS4
GO:0005575	2	6.61E-06	19260466	MAA_06180	-3.64	peroxidase manganese-dependent 1
GO:0005575	2	6.61E-06	19262048	MAA_07762	-3.64	hypothetical protein MAA_07762
GO:0005575	2	6.61E-06	19263074	MAA_08788	-3.60	ankyrin repeat-containing protein
GO:0005575	2	6.61E-06	19261383	MAA_07097	-3.19	C2H2 transcription factor
GO:0005575	2	6.61E-06	19263838	MAA_09552	-3.11	FAD-binding, type 2
GO:0005575	2	6.61E-06	23632897	MAA_11449	-2.96	Ankyrin repeat-containing domain protein
GO:0005575	2	6.61E-06	19255921	MAA_01635	-2.92	integral membrane protein pth11
GO:0005575	2	6.61E-06	19254665	MAA_00379	-2.77	tlh5
GO:0005575	2	6.61E-06	19263667	MAA_09381	-2.75	protoporphyrinogen oxidase
GO:0005575	2	6.61E-06	19263325	MAA_09039	-2.73	hydantoinase
GO:0005575	2	6.61E-06	19264331	MAA_10045	-2.72	Aldo/keto reductase, DtxS3
GO:0005575	2	6.61E-06	19263851	MAA_09565	-2.70	Metallophosphoesterase
GO:0005575	2	6.61E-06	23632304	MAA_10855	-2.66	hypothetical protein MAA_10855
GO:0005575	2	6.61E-06	19261160	MAA_06874	-2.59	Necrosis inducing
GO:0005575	2	6.61E-06	19262950	MAA_08664	-2.47	hypothetical protein MAA_08664
GO:0005575	2	6.61E-06	19259811	MAA_05525	-2.46	endo-1,3(4)-beta-glucanase
GO:0005575	2	6.61E-06	19257223	MAA_02937	-2.41	hypothetical protein MAA_02937
GO:0005575	2	6.61E-06	19262575	MAA_08289	-2.35	cell surface protein (Mas1)
GO:0005575	2	6.61E-06	19257525	MAA_03239	-2.33	Beta-ketoacyl synthase
GO:0005575	2	6.61E-06	19263547	MAA_09261	-2.25	Regulator of G protein signaling superfamily
GO:0005575	2	6.61E-06	19264670	MAA_10384	-2.20	dynamin family protein
GO:0005575	2	6.61E-06	19262060	MAA_07774	-2.19	ankyrin protein
GO:0005575	2	6.61E-06	19264754	MAA_10468	-2.18	catalytic protein
GO:0005575	2	6.61E-06	19254299	MAA_00013	-2.17	lipase, class 3
GO:0005575	2	6.61E-06	19256264	MAA_01978	-2.14	hypothetical protein MAA_01978
GO:0005575	2	6.61E-06	19264234	MAA_09948	-2.13	nitroreductase protein
GO:0005575	2	6.61E-06	19261774	MAA_07488	-2.11	exo-1,3-beta-D-glucanase
GO:0005575	2	6.61E-06	19259153	MAA_04867	-2.08	Pyridine nucleotide-disulfide oxidoreductase
GO:0005575	2	6.61E-06	19262292	MAA_08006	-1.99	hypothetical protein MAA_08006
GO:0005575	2	6.61E-06	19259894	MAA_05608	-1.98	hypothetical protein MAA_05608
GO:0005575	2	6.61E-06	19254732	MAA_00446	-1.97	NADPH dehydrogenase
GO:0005575	2	6.61E-06	19260857	MAA_06571	-1.95	NAD(P)-binding domain protein
GO:0005575	2	6.61E-06	19256668	MAA_02382	-1.91	Oligopeptide transporter OPT superfamily
GO:0005575	2	6.61E-06	19258919	MAA_04633	-1.90	hypothetical protein MAA_04633
GO:0005575	2	6.61E-06	19264518	MAA_10232	-1.88	ankyrin repeat containing protein
GO:0005575	2	6.61E-06	19256825	MAA_02539	-1.86	cell surface protein (Mas1)
GO:0005575	2	6.61E-06	19261648	MAA_07362	-1.85	RNA-directed RNA polymerase (Sad-1)
GO:0005575	2	6.61E-06	19257527	MAA_03241	-1.82	glutamyl-tRNA(Gln) amidotransferase
GO:0005575	2	6.61E-06	19264330	MAA_10044	-1.78	Cytochrome P450, DtxS2
GO:0005575	2	6.61E-06	19261778	MAA_07492	-1.78	c6 zinc finger domain containing protein
GO:0005575	2	6.61E-06	19263736	MAA_09450	-1.76	proteinrelated to ser/arg-related nuclear matrix protein

GO:0005575	2	6.61E-06	19264203	MAA_09917	-1.74	Cellular retinaldehyde-binding/triple function
GO:0005575	2	6.61E-06	23633221	MAA_11773	-1.72	hypothetical protein MAA_11773
GO:0005575	2	6.61E-06	19254338	MAA_00052	-1.71	Nucleoside phosphorylase domain protein
GO:0005575	2	6.61E-06	19256493	MAA_02207	-1.71	carbohydrate esterase family 9 protein
GO:0005575	2	6.61E-06	19254288	MAA_00002	-1.67	hypothetical protein MAA_00002
GO:0005575	2	6.61E-06	19255591	MAA_01305	-1.67	hypothetical protein MAA_01305
GO:0005575	2	6.61E-06	19263800	MAA_09514	-1.67	Cytochrome P450 CYP58S1
GO:0005575	2	6.61E-06	19259418	MAA_05132	-1.66	NAD(P)-binding protein
GO:0005575	2	6.61E-06	19255592	MAA_01306	-1.64	protein kinase-like domain protein
GO:0005575	2	6.61E-06	19255540	MAA_01254	-1.63	hypothetical protein X797_005849
GO:0005575	2	6.61E-06	19263305	MAA_09019	-1.62	tri14-like protein
GO:0005575	2	6.61E-06	19255287	MAA_01001	-1.61	GCN5-related N-acetyltransferase (GNAT) domain protein
GO:0005575	2	6.61E-06	19257988	MAA_03702	-1.61	transcription factor RfeG
GO:0005575	2	6.61E-06	19261793	MAA_07507	-1.59	Pyoverdine biosynthesis
GO:0005575	2	6.61E-06	19259509	MAA_05223	-1.56	CAS1 protein
GO:0005575	2	6.61E-06	19254343	MAA_00057	-1.53	choline dehydrogenase
GO:0005575	2	6.61E-06	19261230	MAA_06944	-1.53	UDP-glucose 4-epimerase
GO:0005575	2	6.61E-06	19258281	MAA_03995	-1.51	ThiJ/PfpI family protein
GO:0005575	2	6.61E-06	19264329	MAA_10043	-1.49	destruxin synthetase, DtxS1
GO:0005575	2	6.61E-06	19263759	MAA_09473	-1.48	DUF1338 domain protein
GO:0005575	2	6.61E-06	19255901	MAA_01615	-1.48	glucose oxidase
GO:0005575	2	6.61E-06	19257557	MAA_03271	-1.47	flavin-binding monooxygenase
GO:0005575	2	6.61E-06	19257582	MAA_03296	-1.47	hypothetical protein MAA_03296
GO:0005575	2	6.61E-06	19263128	MAA_08842	-1.45	NAD(P)-binding domain protein
GO:0005575	2	6.61E-06	19254740	MAA_00454	-1.44	Phospholipase C, phosphatidylinositol-specific, X domain protein
GO:0005575	2	6.61E-06	19263514	MAA_09228	-1.40	lysozyme
GO:0005575	2	6.61E-06	19260070	MAA_05784	-1.39	hypothetical protein MAA_05784
GO:0005575	2	6.61E-06	19254364	MAA_00078	-1.39	FAD dependent monooxygenase
GO:0005575	2	6.61E-06	19261013	MAA_06727	-1.39	duf614 domain containing protein
GO:0005575	2	6.61E-06	19264752	MAA_10466	-1.39	Trimeric LpxA-like protein
GO:0005575	2	6.61E-06	19257586	MAA_03300	-1.37	Tetratricopeptide repeat protein
GO:0005575	2	6.61E-06	19261981	MAA_07695	-1.37	mitochondrial chaperone BCS1
GO:0005575	2	6.61E-06	19259921	MAA_05635	-1.37	CsbD-like protein
GO:0005575	2	6.61E-06	19256461	MAA_02175	-1.36	immunoglobulin I-set domain-containing protein
GO:0005575	2	6.61E-06	23632740	MAA_11292	-1.36	hypothetical protein MAA_11292
GO:0005575	2	6.61E-06	19261596	MAA_07310	-1.34	integral membrane family protein
GO:0005575	2	6.61E-06	19261280	MAA_06994	-1.33	secretory lipase
GO:0005575	2	6.61E-06	19257069	MAA_02783	-1.32	mucoidy inhibitor A
GO:0005575	2	6.61E-06	19255482	MAA_01196	-1.32	cysteine-rich secreted protein
GO:0005575	2	6.61E-06	19262758	MAA_08472	-1.32	Glycerate kinase
GO:0005575	2	6.61E-06	19262103	MAA_07817	-1.31	glycoside hydrolase family 76 protein
GO:0005575	2	6.61E-06	19259756	MAA_05470	-1.30	transferase family protein
GO:0005575	2	6.61E-06	19257345	MAA_03059	-1.29	meiotically up-regulated 65 protein
GO:0005575	2	6.61E-06	19262044	MAA_07758	-1.29	coagulation factor 5/8 type domain protein
GO:0005575	2	6.61E-06	19259139	MAA_04853	-1.28	hypothetical protein MAA_04853
GO:0005575	2	6.61E-06	19261242	MAA_06956	-1.26	hypothetical protein MAA_06956
GO:0005575	2	6.61E-06	19259557	MAA_05271	-1.24	nicotinamide N-methyltransferase
GO:0005575	2	6.61E-06	19261083	MAA_06797	-1.24	peptidase family M28
GO:0005575	2	6.61E-06	19262418	MAA_08132	-1.24	hypothetical protein MAA_08132
GO:0005575	2	6.61E-06	19258721	MAA_04435	-1.23	Salicylate synthase
GO:0005575	2	6.61E-06	19264231	MAA_09945	-1.22	c6 zinc finger domain containing protein
GO:0005575	2	6.61E-06	19256757	MAA_02471	-1.22	phosducin
GO:0005575	2	6.61E-06	19263892	MAA_09606	-1.21	FAD binding domain-containing protein
GO:0005575	2	6.61E-06	19263958	MAA_09672	-1.20	WD domain-containing protein
GO:0005575	2	6.61E-06	19258805	MAA_04519	-1.19	caspase domain-containing protein
GO:0005575	2	6.61E-06	19255970	MAA_01684	-1.19	Leucine Rich Repeat domain protein
GO:0005575	2	6.61E-06	19263848	MAA_09562	-1.18	fatty acid hydroxylase superfamily protein
GO:0005575	2	6.61E-06	19261382	MAA_07096	-1.18	hypothetical protein MAA_07096
GO:0005575	2	6.61E-06	19257045	MAA_02759	-1.18	Ca2+ permeable channel
GO:0005575	2	6.61E-06	19258127	MAA_03841	-1.18	Cytochrome P450 CYP6003A1
GO:0005575	2	6.61E-06	19264076	MAA_09790	-1.16	hypothetical protein MAA_09790

GO:0005575	2	6.61E-06	19258535	MAA_04249	-1.16	methyltransferase
GO:0005575	2	6.61E-06	19258280	MAA_03994	-1.15	glycosyl transferase GT-A type structural fold protein
GO:0005575	2	6.61E-06	19258514	MAA_04228	-1.15	phospho-2-dehydro-3-deoxyheptonate aldolase
GO:0005575	2	6.61E-06	19262192	MAA_07906	-1.15	Alpha-hydroxy acid dehydrogenase, FMN-dependent
GO:0005575	2	6.61E-06	23632203	MAA_10754	-1.15	gag-pol polyprotein
GO:0005575	2	6.61E-06	19255594	MAA_01308	-1.14	Peptidase S8/S53, subtilisin/kexin/sedolisin
GO:0005575	2	6.61E-06	19261293	MAA_07007	-1.14	alkaline phosphatase-like protein
GO:0005575	2	6.61E-06	19255473	MAA_01187	-1.13	DUF2205 domain protein [Metarhizium robertsii]
GO:0005575	2	6.61E-06	19264476	MAA_10190	-1.12	Heterokaryon incompatibility
GO:0005575	2	6.61E-06	19263310	MAA_09024	-1.11	selenium-binding protein
GO:0005575	2	6.61E-06	19254358	MAA_00072	-1.10	hypothetical protein MAA_00072
GO:0005575	2	6.61E-06	19261473	MAA_07187	-1.09	expression library immunization antigen 1
GO:0005575	2	6.61E-06	19262532	MAA_08246	-1.09	ankyrin repeat domain containing protein
GO:0005575	2	6.61E-06	19260886	MAA_06600	-1.09	hypothetical protein MAA_06600
GO:0005575	2	6.61E-06	19257500	MAA_03214	-1.08	Amino acid transporter, transmembrane
GO:0005575	2	6.61E-06	19261097	MAA_06811	-1.07	hypothetical protein H634G_06914
GO:0005575	2	6.61E-06	19255698	MAA_01412	-1.06	Ankyrin repeat-containing domain protein
GO:0005575	2	6.61E-06	19263194	MAA_08908	-1.06	FHA domain containing protein
GO:0005575	2	6.61E-06	19261231	MAA_06945	-1.05	glycosyltransferase family 4 protein
GO:0005575	2	6.61E-06	19256410	MAA_02124	-1.05	menaquinone methyltransferase related to dlpA protein
GO:0005575	2	6.61E-06	19262238	MAA_07952	-1.03	Glycosyl transferase, family 25
GO:0005575	2	6.61E-06	23632270	MAA_10821	-1.03	ubiquitin-conjugating enzyme
GO:0005575	2	6.61E-06	19262129	MAA_07843	-1.02	patatin-like phospholipase
GO:0005575	2	6.61E-06	19258332	MAA_04046	-1.02	RNA recognition motif domain protein
GO:0005575	2	6.61E-06	19256214	MAA_01928	-1.02	Ubiquitin 3 binding protein But2
GO:0005575	2	6.61E-06	19255412	MAA_01126	-1.02	Alpha/beta hydrolase fold-3
GO:0005575	2	6.61E-06	19255071	MAA_00785	-1.02	annexin ANXC4
GO:0005575	2	6.61E-06	19261480	MAA_07194	-1.01	hypothetical protein MAA_07194
GO:0005575	2	6.61E-06	19264829	MAA_10543	1.00	aminoglycoside 3'-phosphotransferase/choline kinase domain protein
GO:0005575	2	6.61E-06	19255584	MAA_01298	1.01	O-methyltransferase
GO:0005575	2	6.61E-06	19261146	MAA_06860	1.01	F-box domain protein
GO:0005575	2	6.61E-06	19260841	MAA_06555	1.02	methyltransferase
GO:0005575	2	6.61E-06	19263890	MAA_09604	1.02	Ig domain protein group 2 domain protein
GO:0005575	2	6.61E-06	19263417	MAA_09131	1.04	zinc finger domain-containing protein
GO:0005575	2	6.61E-06	23633159	MAA_11711	1.04	zinc finger domain-containing protein
GO:0005575	2	6.61E-06	19263978	MAA_09692	1.04	Oxoglutarate/iron-dependent oxygenase
GO:0005575	2	6.61E-06	19256915	MAA_02629	1.05	Alcohol dehydrogenase superfamily, zinc-type
GO:0005575	2	6.61E-06	19263391	MAA_09105	1.06	hypothetical protein MAA_09105
GO:0005575	2	6.61E-06	19262673	MAA_08387	1.06	short-chain dehydrogenase
GO:0005575	2	6.61E-06	19262471	MAA_08185	1.06	LEA domain protein
GO:0005575	2	6.61E-06	19255677	MAA_01391	1.06	transposase
GO:0005575	2	6.61E-06	19259242	MAA_04956	1.07	Amine oxidase
GO:0005575	2	6.61E-06	19262964	MAA_08678	1.07	hypothetical protein MAA_08678
GO:0005575	2	6.61E-06	23632906	MAA_11458	1.07	hypothetical protein MAA_11458
GO:0005575	2	6.61E-06	19257788	MAA_03502	1.09	zeta toxin family protein
GO:0005575	2	6.61E-06	19262692	MAA_08406	1.10	glycoside hydrolase family 16
GO:0005575	2	6.61E-06	19256318	MAA_02032	1.11	3-phytase
GO:0005575	2	6.61E-06	19263423	MAA_09137	1.12	Protein kinase-like protein
GO:0005575	2	6.61E-06	19264721	MAA_10435	1.12	sulfite oxidase
GO:0005575	2	6.61E-06	19259511	MAA_05225	1.12	extracellular dipeptidyl-peptidase Dpp4
GO:0005575	2	6.61E-06	19258466	MAA_04180	1.12	TfdA family Taurine catabolism dioxygenase TauD
GO:0005575	2	6.61E-06	19258184	MAA_03898	1.14	long-chain-fatty-acid-CoA ligase
GO:0005575	2	6.61E-06	19255889	MAA_01603	1.14	hypothetical protein MAA_01603
GO:0005575	2	6.61E-06	19264450	MAA_10164	1.14	reverse transcriptase, RNaseH
GO:0005575	2	6.61E-06	19263386	MAA_09100	1.14	DDE superfamily endonuclease, CENP-B-like protein
GO:0005575	2	6.61E-06	19257778	MAA_03492	1.14	feruloyl esterase B
GO:0005575	2	6.61E-06	19262472	MAA_08186	1.14	late embryogenesis abundant protein
GO:0005575	2	6.61E-06	19258564	MAA_04278	1.15	hypothetical protein MAA_04278
GO:0005575	2	6.61E-06	19264024	MAA_09738	1.15	ThiJ/PfpI family protein
GO:0005575	2	6.61E-06	19263888	MAA_09602	1.16	Six-hairpin glycosidase
GO:0005575	2	6.61E-06	19263266	MAA_08980	1.16	Cyanovirin-N

GO:0005575	2	6.61E-06	19263977	MAA_09691	1.17	1-aminocyclopropane-1-carboxylate synthase
GO:0005575	2	6.61E-06	19260840	MAA_06554	1.19	delta-12 fatty acid desaturase
GO:0005575	2	6.61E-06	19260584	MAA_06298	1.19	MFS transporter [Metarhizium robertsii]
GO:0005575	2	6.61E-06	19263497	MAA_09211	1.19	hypothetical protein MAA_09211
GO:0005575	2	6.61E-06	19257789	MAA_03503	1.19	Protein kinase-like protein
GO:0005575	2	6.61E-06	19264772	MAA_10486	1.20	gamma-glutamyltranspeptidase periplasmic precursor
GO:0005575	2	6.61E-06	19260689	MAA_06403	1.21	glycoside hydrolase family 76 protein
GO:0005575	2	6.61E-06	19264141	MAA_09855	1.22	4-hydroxyphenylpyruvate dioxygenase
GO:0005575	2	6.61E-06	19259110	MAA_04824	1.23	non-ribosomal peptide synthetase
GO:0005575	2	6.61E-06	19264818	MAA_10532	1.23	peptidase family M3
GO:0005575	2	6.61E-06	19257096	MAA_02810	1.24	Fungal chitosanase
GO:0005575	2	6.61E-06	19264758	MAA_10472	1.26	gag-pol polyprotein
GO:0005575	2	6.61E-06	19255997	MAA_01711	1.27	Beta-lactamase-like protein
GO:0005575	2	6.61E-06	19258830	MAA_04544	1.27	hypothetical protein MAA_04544
GO:0005575	2	6.61E-06	19260869	MAA_06583	1.29	feruloyl esterase B
GO:0005575	2	6.61E-06	19264070	MAA_09784	1.34	16 kDa allergen
GO:0005575	2	6.61E-06	19255583	MAA_01297	1.38	haloacid dehalogenase
GO:0005575	2	6.61E-06	19260837	MAA_06551	1.39	macrophomate synthase
GO:0005575	2	6.61E-06	23632196	MAA_10747	1.39	ankyrin repeat protein
GO:0005575	2	6.61E-06	23632364	MAA_10915	1.41	hypothetical protein MAA_10915
GO:0005575	2	6.61E-06	19263023	MAA_08737	1.42	Beta-lactamase-like protein
GO:0005575	2	6.61E-06	19254392	MAA_00106	1.44	F-box domain protein
GO:0005575	2	6.61E-06	19264325	MAA_10039	1.44	subgroup IIIi aminotransferase
GO:0005575	2	6.61E-06	19263202	MAA_08916	1.45	multicopper oxidase
GO:0005575	2	6.61E-06	19254406	MAA_00120	1.45	Methyltransferase type 11
GO:0005575	2	6.61E-06	19264066	MAA_09780	1.45	glycosyl transferase, group 2 family protein
GO:0005575	2	6.61E-06	19260260	MAA_05974	1.46	S-adenosylmethionine:tRNA ribosyltransferase-isomerase
GO:0005575	2	6.61E-06	19264827	MAA_10541	1.47	hypothetical protein MAA_10541
GO:0005575	2	6.61E-06	19263587	MAA_09301	1.49	F-box domain-containing protein
GO:0005575	2	6.61E-06	19262651	MAA_08365	1.51	multicopper oxidase
GO:0005575	2	6.61E-06	19258327	MAA_04041	1.61	hypothetical protein MAA_04041
GO:0005575	2	6.61E-06	19256546	MAA_02260	1.62	sarcosine oxidase
GO:0005575	2	6.61E-06	19260261	MAA_05975	1.62	(2R)-phospho-3-sulfolactate synthase, ComA
GO:0005575	2	6.61E-06	19263904	MAA_09618	1.65	CobW domain protein
GO:0005575	2	6.61E-06	19264611	MAA_10325	1.69	Nitrilase/cyanide hydratase and apolipoprotein N-acyltransferase
GO:0005575	2	6.61E-06	19255639	MAA_01353	1.69	Oxoglutarate/iron-dependent oxygenase
GO:0005575	2	6.61E-06	23633070	MAA_11622	1.71	hypothetical protein MAA_11622
GO:0005575	2	6.61E-06	19264142	MAA_09856	1.72	hypothetical protein MAA_09856
GO:0005575	2	6.61E-06	19264386	MAA_10100	1.72	hypothetical protein MAA_10100
GO:0005575	2	6.61E-06	19262890	MAA_08604	1.74	hypothetical protein MAA_08604
GO:0005575	2	6.61E-06	19258065	MAA_03779	1.79	peptidylarginine deiminase-like enzyme
GO:0005575	2	6.61E-06	19262652	MAA_08366	1.80	methyltransferase [Metarhizium robertsii]
GO:0005575	2	6.61E-06	19262904	MAA_08618	1.81	Phytanoyl-CoA dioxygenase PhyH
GO:0005575	2	6.61E-06	19255194	MAA_00908	1.81	might be a transmembrane protein
GO:0005575	2	6.61E-06	19263545	MAA_09259	1.84	epoxidase subunit A
GO:0005575	2	6.61E-06	19262906	MAA_08620	1.84	nmrA-like family protein
GO:0005575	2	6.61E-06	19255954	MAA_01668	1.84	Phosphoesterase
GO:0005575	2	6.61E-06	19262903	MAA_08617	1.87	aromatic amino acid aminotransferase
GO:0005575	2	6.61E-06	19263645	MAA_09359	1.89	Pfs, NACHT and WD domain protein
GO:0005575	2	6.61E-06	19263747	MAA_09461	1.93	carbohydrate-binding WSC domain protein
GO:0005575	2	6.61E-06	19257622	MAA_03336	1.94	Peptidase S8/S53, subtilisin/kexin/sedolisin
GO:0005575	2	6.61E-06	19262577	MAA_08291	1.94	sulfatase domain-containing protein
GO:0005575	2	6.61E-06	19264817	MAA_10531	1.95	Oxoglutarate/iron-dependent oxygenase
GO:0005575	2	6.61E-06	19258293	MAA_04007	1.95	alcohol dehydrogenase GroES domain-containing protein
GO:0005575	2	6.61E-06	19262927	MAA_08641	1.97	sulfatase domain protein
GO:0005575	2	6.61E-06	19264603	MAA_10317	1.98	pyridoxal-dependent decarboxylase domain protein
GO:0005575	2	6.61E-06	19263643	MAA_09357	1.99	hypothetical protein X797_010135
GO:0005575	2	6.61E-06	19262974	MAA_08688	2.00	CFEM domain protein [Metarhizium robertsii]
GO:0005575	2	6.61E-06	19262655	MAA_08369	2.07	Dimeric alpha-beta barrel
GO:0005575	2	6.61E-06	19264481	MAA_10195	2.07	Ctr copper transporter
GO:0005575	2	6.61E-06	19260312	MAA_06026	2.08	phosphatidylserine decarboxylase family protein

GO:0005575	2	6.61E-06	19262909	MAA_08623	2.21	phytanoyl-CoA dioxygenase
GO:0005575	2	6.61E-06	19264319	MAA_10033	2.28	polyketide synthase
GO:0005575	2	6.61E-06	19260992	MAA_06706	2.30	short-chain dehydrogenase
GO:0005575	2	6.61E-06	19262907	MAA_08621	2.31	f420-dependent NADP reductase
GO:0005575	2	6.61E-06	19258452	MAA_04166	2.35	DUF1275 domain protein
GO:0005575	2	6.61E-06	19264505	MAA_10219	2.44	FAD/FMN-containing isoamyl alcohol oxidase MreA
GO:0005575	2	6.61E-06	23633189	MAA_11741	2.75	hypothetical protein MAA_11741
GO:0005575	2	6.61E-06	19258063	MAA_03777	2.87	Bys1 family protein
GO:0005575	2	6.61E-06	19255706	MAA_01420	3.28	hypothetical protein MAA_01420
GO:0005575	2	6.61E-06	19264610	MAA_10324	3.62	NmrA-like family protein
GO:0005575	2	6.61E-06	19255348	MAA_01062	7.81	hypothetical protein MAA_01062
GO:0005575	2	6.61E-06	19264488	MAA_10202	14.39	Beta-ketoacyl synthase
GO:0004252	8	4.41E-05	19263004	MAA_08718	-6.63	Peptidase S8, subtilisin, Asp-active site protein
GO:0004252	8	4.41E-05	19259961	MAA_05675	-4.48	alkaline serine protease P32
GO:0004252	8	4.41E-05	23632468	MAA_11020	-3.77	peptidase S1 domain protein
GO:0004252	8	4.41E-05	19263878	MAA_09592	-3.08	Peptidase S1/S6, chymotrypsin/Hap
GO:0004252	8	4.41E-05	19257548	MAA_03262	-2.40	Peptidase S1/S6, chymotrypsin/Hap
GO:0004252	8	4.41E-05	19262987	MAA_08701	-1.69	subtilisin-like serine protease precursor
GO:0004252	8	4.41E-05	19263922	MAA_09636	-1.21	subtilisin-like protease
GO:0004252	8	4.41E-05	19255594	MAA_01308	-1.14	Peptidase S8/S53, subtilisin/kexin/sedolisin
GO:0004252	8	4.41E-05	19262454	MAA_08168	-1.03	Peptidase S8, subtilisin-related protein
GO:0004252	8	4.41E-05	19254778	MAA_00492	1.28	subtilisin-like protease
GO:0004252	8	4.41E-05	19257622	MAA_03336	1.94	Peptidase S8/S53, subtilisin/kexin/sedolisin
GO:0004252	8	4.41E-05	19262821	MAA_08535	11.37	tripeptidyl peptidase a
GO:0042438	8	6.09E-05	19264708	MAA_10422	-3.24	hypothetical protein MAA_10422
GO:0042438	8	6.09E-05	19257525	MAA_03239	-2.33	Beta-ketoacyl synthase
GO:0042438	8	6.09E-05	19257527	MAA_03241	-1.82	glutamyl-tRNA(Gln) amidotransferase
GO:0042438	8	6.09E-05	19257526	MAA_03240	-3.79	hypothetical protein MAA_03240
GO:0042438	8	6.09E-05	23632196	MAA_10747	1.39	ankyrin repeat protein
GO:0006508	7	1.65E-04	19263004	MAA_08718	-6.63	Peptidase S8, subtilisin, Asp-active site protein
GO:0006508	7	1.65E-04	19263490	MAA_09204	-5.66	Concanavalin A-like lectin/glucanase
GO:0006508	7	1.65E-04	19255283	MAA_00997	-4.09	peptidase family M28
GO:0006508	7	1.65E-04	19255716	MAA_01430	-2.98	Peptidase A1
GO:0006508	7	1.65E-04	19262512	MAA_08226	-2.72	Peptidase aspartic, active site protein
GO:0006508	7	1.65E-04	23632201	MAA_10752	-1.74	triacylglycerol lipase-like protein
GO:0006508	7	1.65E-04	19255800	MAA_01514	-1.18	Peptidase A1
GO:0006508	7	1.65E-04	19255594	MAA_01308	-1.14	Peptidase S8/S53, subtilisin/kexin/sedolisin
GO:0006508	7	1.65E-04	19255772	MAA_01486	1.11	serine carboxypeptidase
GO:0006508	7	1.65E-04	19259511	MAA_05225	1.12	extracellular dipeptidyl-peptidase Dpp4
GO:0006508	7	1.65E-04	19264818	MAA_10532	1.23	peptidase family M3
GO:0006508	7	1.65E-04	19257622	MAA_03336	1.94	Peptidase S8/S53, subtilisin/kexin/sedolisin
GO:0006508	7	1.65E-04	19255985	MAA_01699	2.40	serin endopeptidase
GO:0006508	7	1.65E-04	19262821	MAA_08535	11.37	tripeptidyl peptidase a
GO:0005576	3	4.33E-04	19263004	MAA_08718	-6.63	Peptidase S8, subtilisin, Asp-active site protein
GO:0005576	3	4.33E-04	19263490	MAA_09204	-5.66	Concanavalin A-like lectin/glucanase
GO:0005576	3	4.33E-04	19262598	MAA_08312	-5.18	cell wall protein
GO:0005576	3	4.33E-04	19255283	MAA_00997	-4.09	peptidase family M28
GO:0005576	3	4.33E-04	19260495	MAA_06209	-3.07	Concanavalin A-like lectin/glucanase
GO:0005576	3	4.33E-04	19256036	MAA_01750	-2.42	bacterial-type extracellular deoxyribonuclease
GO:0005576	3	4.33E-04	19262601	MAA_08315	-2.30	Glycoside hydrolase, chitinase active site protein
GO:0005576	3	4.33E-04	19263527	MAA_09241	-2.28	cysteine-rich secretory protein
GO:0005576	3	4.33E-04	19263304	MAA_09018	-1.80	Glycoside hydrolase, subgroup, catalytic core
GO:0005576	3	4.33E-04	19263052	MAA_08766	-1.67	maltase
GO:0005576	3	4.33E-04	19256453	MAA_02167	-1.51	Glucoamylase, putative
GO:0005576	3	4.33E-04	19256971	MAA_02685	-1.44	chitinase
GO:0005576	3	4.33E-04	19259713	MAA_05427	-1.43	LysM domain-containing protein
GO:0005576	3	4.33E-04	19261227	MAA_06941	-1.35	extracellular serine-rich protein
GO:0005576	3	4.33E-04	19254294	MAA_00008	-1.27	Alpha-L-arabinofuranosidase B, catalytic
GO:0005576	3	4.33E-04	19254739	MAA_00453	-1.22	Ribonuclease T2, active site protein
GO:0005576	3	4.33E-04	19255772	MAA_01486	1.11	serine carboxypeptidase
GO:0000977	9	7.88E-04	23633117	MAA_11669	-13.77	hypothetical protein MAA_11669

GO:0000977	9	7.88E-04	19255215	MAA_00929	-2.17	Ribonuclease H-like protein
GO:0000977	9	7.88E-04	23632363	MAA_10914	1.00	pol-like protein
GO:0000977	9	7.88E-04	23633175	MAA_11727	1.08	FluG domain-containing protein
GO:0000977	9	7.88E-04	19254667	MAA_00381	1.11	Ribonuclease H-like protein
GO:0000977	9	7.88E-04	19263392	MAA_09106	1.12	FluG domain-containing protein
GO:0000977	9	7.88E-04	19264446	MAA_10160	1.20	transposase-like protein
GO:0000977	9	7.88E-04	19264853	MAA_10567	1.42	restless-like transposase
GO:0051213	5	8.23E-04	19259775	MAA_05489	-2.03	Oxoglutarate/iron-dependent oxygenase
GO:0051213	5	8.23E-04	19258127	MAA_03841	-1.18	Cytochrome P450 CYP6003A1
GO:0051213	5	8.23E-04	19259541	MAA_05255	-1.14	isopenicillin N synthase-like protein
GO:0051213	5	8.23E-04	19262904	MAA_08618	1.81	Phytanoyl-CoA dioxygenase PhyH
GO:0051213	5	8.23E-04	19262909	MAA_08623	2.21	phytanoyl-CoA dioxygenase
GO:0031177	6	1.10E-03	19261030	MAA_06744	-9.56	lysergyl peptide synthetase subunit 3 LPS3
GO:0031177	6	1.10E-03	19262985	MAA_08699	-8.86	Beta-ketoacyl synthase
GO:0031177	6	1.10E-03	19260861	MAA_06575	-4.95	Beta-ketoacyl synthase
GO:0031177	6	1.10E-03	19257525	MAA_03239	-2.33	Beta-ketoacyl synthase
GO:0031177	6	1.10E-03	23633221	MAA_11773	-1.72	hypothetical protein MAA_11773
GO:0031177	6	1.10E-03	19259756	MAA_05470	-1.30	transferase family protein
GO:0031177	6	1.10E-03	23632196	MAA_10747	1.39	ankyrin repeat protein
GO:0031177	6	1.10E-03	19262652	MAA_08366	1.80	methyltransferase
GO:0019829	8	1.51E-03	19259842	MAA_05556	-2.72	potassium/sodium efflux P-type ATPase
GO:0019829	8	1.51E-03	19264010	MAA_09724	-1.37	ATPase, P-type, potassium/sodium efflux, fungal
GO:0019829	8	1.51E-03	19259102	MAA_04816	-1.08	cation transporting ATPase
GO:0019829	8	1.51E-03	19264538	MAA_10252	-1.02	magnesium-translocating P-type ATPase
GO:0004668	7	1.51E-03	19259268	MAA_04982	-4.36	protein-arginine deiminase type-4
GO:0004668	7	1.51E-03	19262135	MAA_07849	-3.25	protein-arginine deiminase type-4
GO:0004668	7	1.51E-03	19254780	MAA_00494	1.36	protein-arginine deiminase type-4
GO:0004668	7	1.51E-03	19258065	MAA_03779	1.79	peptidylarginine deiminase-like enzyme
GO:0005623	3	2.47E-03	19259268	MAA_04982	-4.36	protein-arginine deiminase type-4
GO:0005623	3	2.47E-03	19254780	MAA_00494	1.36	protein-arginine deiminase type-4
GO:1900815	7	2.62E-03	19260861	MAA_06575	-4.95	Beta-ketoacyl synthase
GO:1900815	7	2.62E-03	19264708	MAA_10422	-3.24	hypothetical protein MAA_10422
GO:1900815	7	2.62E-03	19257527	MAA_03241	-1.82	glutamyl-tRNA(Gln) amidotransferase
GO:1900815	7	2.62E-03	19262652	MAA_08366	1.80	methyltransferase [Metarhizium robertsii]
GO:1900815	7	2.62E-03	19262655	MAA_08369	2.07	Dimeric alpha-beta barrel
GO:1900815	7	2.62E-03	19264488	MAA_10202	14.39	Beta-ketoacyl synthase
GO:0016614	5	3.12E-03	19263838	MAA_09552	-3.11	FAD-binding, type 2
GO:0016614	5	3.12E-03	19254343	MAA_00057	-1.53	choline dehydrogenase
GO:0016614	5	3.12E-03	19255901	MAA_01615	-1.48	glucose oxidase
GO:0016614	5	3.12E-03	23632270	MAA_10821	-1.03	ubiquitin-conjugating enzyme
GO:0016614	5	3.12E-03	19264018	MAA_09732	1.02	Aldo/keto reductase
GO:0006357	9	3.45E-03	23633117	MAA_11669	-13.77	hypothetical protein MAA_11669
GO:0006357	9	3.45E-03	23633036	MAA_11588	-9.96	SET domain protein
GO:0006357	9	3.45E-03	19255215	MAA_00929	-2.17	Ribonuclease H-like protein
GO:0006357	9	3.45E-03	19260324	MAA_06038	-1.57	pH regulatory protein mrPacC
GO:0006357	9	3.45E-03	23632363	MAA_10914	1.00	pol-like protein
GO:0006357	9	3.45E-03	23633175	MAA_11727	1.08	FluG domain-containing protein
GO:0006357	9	3.45E-03	19254667	MAA_00381	1.11	Ribonuclease H-like protein
GO:0006357	9	3.45E-03	19263392	MAA_09106	1.12	FluG domain-containing protein
GO:0006357	9	3.45E-03	19264446	MAA_10160	1.20	transposase-like protein
GO:0006357	9	3.45E-03	19264853	MAA_10567	1.42	restless-like transposase
GO:0004190	8	3.73E-03	19263490	MAA_09204	-5.66	Concanavalin A-like lectin/glucanase
GO:0004190	8	3.73E-03	19260495	MAA_06209	-3.07	Concanavalin A-like lectin/glucanase
GO:0004190	8	3.73E-03	19255716	MAA_01430	-2.98	Peptidase A1
GO:0004190	8	3.73E-03	19262512	MAA_08226	-2.72	Peptidase aspartic, active site protein
GO:0004190	8	3.73E-03	23632201	MAA_10752	-1.74	triacylglycerol lipase-like protein
GO:0004190	8	3.73E-03	19255800	MAA_01514	-1.18	Peptidase A1
GO:0016747	6	3.85E-03	19262985	MAA_08699	-8.86	Beta-ketoacyl synthase
GO:0016747	6	3.85E-03	23633221	MAA_11773	-1.72	hypothetical protein MAA_11773
GO:0016747	6	3.85E-03	19259418	MAA_05132	-1.66	NAD(P)-binding protein
GO:0016747	6	3.85E-03	19259756	MAA_05470	-1.30	transferase family protein

GO:0016747	6	3.85E-03	19263692	MAA_09406	1.17	hypothetical protein MAA_09406
GO:0016747	6	3.85E-03	19258293	MAA_04007	1.95	alcohol dehydrogenase GroES domain-containing protein
GO:0016747	6	3.85E-03	19264319	MAA_10033	2.28	polyketide synthase
GO:0016747	6	3.85E-03	19264488	MAA_10202	14.39	Beta-ketoacyl synthase
GO:0008236	6	4.06E-03	19255594	MAA_01308	-1.14	Peptidase S8/S53, subtilisin/kexin/sedolisin
GO:0008236	6	4.06E-03	19259511	MAA_05225	1.12	extracellular dipeptidyl-peptidase Dpp4
GO:0008236	6	4.06E-03	19257622	MAA_03336	1.94	Peptidase S8/S53, subtilisin/kexin/sedolisin
GO:0008236	6	4.06E-03	19262821	MAA_08535	11.37	tripeptidyl peptidase a
GO:0003824	3	4.39E-03	19261030	MAA_06744	-9.56	lysergyl peptide synthetase subunit 3 LPS3
GO:0003824	3	4.39E-03	19262985	MAA_08699	-8.86	Beta-ketoacyl synthase
GO:0003824	3	4.39E-03	19262115	MAA_07829	-4.38	WD40 repeat-like-containing domain protein
GO:0003824	3	4.39E-03	19263074	MAA_08788	-3.60	ankyrin repeat-containing protein
GO:0003824	3	4.39E-03	19254732	MAA_00446	-1.97	NADPH dehydrogenase
GO:0003824	3	4.39E-03	19264518	MAA_10232	-1.88	ankyrin repeat containing protein
GO:0003824	3	4.39E-03	23633221	MAA_11773	-1.72	hypothetical protein MAA_11773
GO:0003824	3	4.39E-03	19254338	MAA_00052	-1.71	Nucleoside phosphorylase domain protein
GO:0003824	3	4.39E-03	19264674	MAA_10388	-1.57	Glyoxalase/bleomycin resistance protein/dioxygenase
GO:0003824	3	4.39E-03	19264329	MAA_10043	-1.49	destruxin synthetase, DtxS1
GO:0003824	3	4.39E-03	19259756	MAA_05470	-1.30	transferase family protein
GO:0003824	3	4.39E-03	19262192	MAA_07906	-1.15	Alpha-hydroxy acid dehydrogenase, FMN-dependent
GO:0003824	3	4.39E-03	19255698	MAA_01412	-1.06	Ankyrin repeat-containing domain protein
GO:0003824	3	4.39E-03	19263888	MAA_09602	1.16	Six-hairpin glycosidase
GO:0003824	3	4.39E-03	19263977	MAA_09691	1.17	1-aminocyclopropane-1-carboxylate synthase
GO:0003824	3	4.39E-03	19259110	MAA_04824	1.23	non-ribosomal peptide synthetase
GO:0003824	3	4.39E-03	19260837	MAA_06551	1.39	macrophomate synthase
GO:0003824	3	4.39E-03	19264325	MAA_10039	1.44	subgroup IIIi aminotransferase
GO:0003824	3	4.39E-03	19260261	MAA_05975	1.62	(2R)-phospho-3-sulfolactate synthase, ComA
GO:0003824	3	4.39E-03	19262652	MAA_08366	1.80	methyltransferase [Metarhizium robertsii]
GO:0003824	3	4.39E-03	19262903	MAA_08617	1.87	aromatic amino acid aminotransferase
GO:0003824	3	4.39E-03	19262577	MAA_08291	1.94	sulfatase domain-containing protein
GO:0003824	3	4.39E-03	19262927	MAA_08641	1.97	sulfatase domain protein
GO:0003824	3	4.39E-03	19264603	MAA_10317	1.98	pyridoxal-dependent decarboxylase domain protein
GO:0003824	3	4.39E-03	19264319	MAA_10033	2.28	polyketide synthase
GO:0003824	3	4.39E-03	19264488	MAA_10202	14.39	Beta-ketoacyl synthase
GO:0019748	4	4.61E-03	19261030	MAA_06744	-9.56	lysergyl peptide synthetase subunit 3 LPS3
GO:0019748	4	4.61E-03	19262985	MAA_08699	-8.86	Beta-ketoacyl synthase
GO:0019748	4	4.61E-03	19260861	MAA_06575	-4.95	Beta-ketoacyl synthase
GO:0019748	4	4.61E-03	23633221	MAA_11773	-1.72	hypothetical protein MAA_11773
GO:0019748	4	4.61E-03	19264329	MAA_10043	-1.49	destruxin synthetase, DtxS1
GO:0019748	4	4.61E-03	19259541	MAA_05255	-1.14	isopenicillin N synthase-like protein
GO:0019748	4	4.61E-03	23632270	MAA_10821	-1.03	ubiquitin-conjugating enzyme
GO:0019748	4	4.61E-03	19263169	MAA_08883	1.31	FAD binding domain-containing protein
GO:0019748	4	4.61E-03	19264319	MAA_10033	2.28	polyketide synthase
GO:0019748	4	4.61E-03	19264488	MAA_10202	14.39	Beta-ketoacyl synthase
GO:0005615	4	4.76E-03	19255619	MAA_01333	-4.06	carboxypeptidase, partial
GO:0005615	4	4.76E-03	19256231	MAA_01945	-3.30	fungalysin metallopeptidase
GO:0005615	4	4.76E-03	19263527	MAA_09241	-2.28	cysteine-rich secretory protein
GO:0005615	4	4.76E-03	19262271	MAA_07985	-1.09	carboxylesterase/lipase domain protein
GO:0005615	4	4.76E-03	19262240	MAA_07954	1.31	hypothetical protein MAA_07954
GO:2001307	7	5.67E-03	19255257	MAA_00971	-4.81	Aromatic prenyltransferase, DMATS type
GO:2001307	7	5.67E-03	19257527	MAA_03241	-1.82	glutamyl-tRNA(Gln) amidotransferase
GO:2001307	7	5.67E-03	19264708	MAA_10422	-3.24	hypothetical protein MAA_10422
GO:0016740	4	6.11E-03	19262985	MAA_08699	-8.86	Beta-ketoacyl synthase
GO:0016740	4	6.11E-03	19257525	MAA_03239	-2.33	Beta-ketoacyl synthase
GO:0016740	4	6.11E-03	23633221	MAA_11773	-1.72	hypothetical protein MAA_11773
GO:0016740	4	6.11E-03	23632196	MAA_10747	1.39	ankyrin repeat protein
GO:0016740	4	6.11E-03	19260260	MAA_05974	1.46	S-adenosylmethionine:tRNA ribosyltransferase-isomerase
GO:0016740	4	6.11E-03	19262652	MAA_08366	1.80	methyltransferase [Metarhizium robertsii]
GO:0016740	4	6.11E-03	19264319	MAA_10033	2.28	polyketide synthase
GO:0016740	4	6.11E-03	19264488	MAA_10202	14.39	Beta-ketoacyl synthase
GO:0055114	4	6.63E-03	19262985	MAA_08699	-8.86	Beta-ketoacyl synthase

GO:0055114	4	6.63E-03	19263838	MAA_09552	-3.11	FAD-binding, type 2
GO:0055114	4	6.63E-03	23632897	MAA_11449	-2.96	Ankyrin repeat-containing domain protein
GO:0055114	4	6.63E-03	19259153	MAA_04867	-2.08	Pyridine nucleotide-disulfide oxidoreductase
GO:0055114	4	6.63E-03	19254732	MAA_00446	-1.97	NADPH dehydrogenase
GO:0055114	4	6.63E-03	19264330	MAA_10044	-1.78	Cytochrome P450, DtxS2
GO:0055114	4	6.63E-03	19261778	MAA_07492	-1.78	c6 zinc finger domain containing protein
GO:0055114	4	6.63E-03	23633221	MAA_11773	-1.72	hypothetical protein MAA_11773
GO:0055114	4	6.63E-03	19263800	MAA_09514	-1.67	Cytochrome P450 CYP58S1
GO:0055114	4	6.63E-03	19259418	MAA_05132	-1.66	NAD(P)-binding protein
GO:0055114	4	6.63E-03	19261793	MAA_07507	-1.59	Pyoverdine biosynthesis
GO:0055114	4	6.63E-03	19254343	MAA_00057	-1.53	choline dehydrogenase
GO:0055114	4	6.63E-03	19255901	MAA_01615	-1.48	glucose oxidase
GO:0055114	4	6.63E-03	19263128	MAA_08842	-1.45	NAD(P)-binding domain protein
GO:0055114	4	6.63E-03	19263848	MAA_09562	-1.18	fatty acid hydroxylase superfamily protein
GO:0055114	4	6.63E-03	19258127	MAA_03841	-1.18	Cytochrome P450 CYP6003A1
GO:0055114	4	6.63E-03	23632270	MAA_10821	-1.03	ubiquitin-conjugating enzyme
GO:0055114	4	6.63E-03	19263978	MAA_09692	1.04	Oxoglutarate/iron-dependent oxygenase
GO:0055114	4	6.63E-03	19259242	MAA_04956	1.07	Amine oxidase
GO:0055114	4	6.63E-03	19262824	MAA_08538	1.13	3-hydroxyacid dehydrogenase/reductase
GO:0055114	4	6.63E-03	19263692	MAA_09406	1.17	hypothetical protein MAA_09406
GO:0055114	4	6.63E-03	19264577	MAA_10291	1.32	cyclopentanone 1,2-monooxygenase
GO:0055114	4	6.63E-03	19261170	MAA_06884	1.36	Aldehyde dehydrogenase
GO:0055114	4	6.63E-03	19263202	MAA_08916	1.45	multicopper oxidase
GO:0055114	4	6.63E-03	19256546	MAA_02260	1.62	sarcosine oxidase
GO:0055114	4	6.63E-03	19255639	MAA_01353	1.69	Oxoglutarate/iron-dependent oxygenase
GO:0055114	4	6.63E-03	19263874	MAA_09588	1.89	TfdA family Taurine catabolism dioxygenase TauD
GO:0055114	4	6.63E-03	19264817	MAA_10531	1.95	Oxoglutarate/iron-dependent oxygenase
GO:0055114	4	6.63E-03	19258293	MAA_04007	1.95	alcohol dehydrogenase GroES domain-containing protein
GO:0055114	4	6.63E-03	19262218	MAA_07932	2.17	Pyruvate/2-oxoglutarate dehydrogenase
GO:0055114	4	6.63E-03	19264319	MAA_10033	2.28	polyketide synthase
GO:0055114	4	6.63E-03	19264505	MAA_10219	2.44	FAD/FMN-containing isoamyl alcohol oxidase MreA
GO:0051974	8	6.92E-03	23633205	MAA_11757	1.07	hypothetical protein MAA_11757
GO:0032211	12	6.92E-03	23633205	MAA_11757	1.07	hypothetical protein MAA_11757
GO:0051974	8	6.92E-03	19263390	MAA_09104	1.10	aaa ATPase
GO:0032211	12	6.92E-03	19263390	MAA_09104	1.10	aaa ATPase
GO:0051974	8	6.92E-03	19264434	MAA_10148	1.13	ATP-dependent DNA helicase PIF1
GO:0032211	12	6.92E-03	19264434	MAA_10148	1.13	ATP-dependent DNA helicase PIF1
GO:0051974	8	6.92E-03	19264635	MAA_10349	1.28	ATP-dependent DNA helicase PIF1
GO:0032211	12	6.92E-03	19264635	MAA_10349	1.28	ATP-dependent DNA helicase PIF1
GO:0051974	8	6.92E-03	19254648	MAA_00362	1.35	PIF1 protein
GO:0032211	12	6.92E-03	19254648	MAA_00362	1.35	PIF1 protein
GO:0044806	9	6.92E-03	23633205	MAA_11757	1.07	hypothetical protein MAA_11757
GO:0044806	9	6.92E-03	19263390	MAA_09104	1.10	aaa ATPase
GO:0044806	9	6.92E-03	19264434	MAA_10148	1.13	ATP-dependent DNA helicase PIF1
GO:0044806	9	6.92E-03	19264635	MAA_10349	1.28	ATP-dependent DNA helicase PIF1
GO:0044806	9	6.92E-03	19254648	MAA_00362	1.35	PIF1 protein
GO:0016491	4	7.11E-03	19262985	MAA_08699	-8.86	Beta-ketoacyl synthase
GO:0016491	4	7.11E-03	23632897	MAA_11449	-2.96	Ankyrin repeat-containing domain protein
GO:0016491	4	7.11E-03	19264234	MAA_09948	-2.13	nitroreductase protein
GO:0016491	4	7.11E-03	19259153	MAA_04867	-2.08	Pyridine nucleotide-disulfide oxidoreductase
GO:0016491	4	7.11E-03	19254732	MAA_00446	-1.97	NADPH dehydrogenase
GO:0016491	4	7.11E-03	19260857	MAA_06571	-1.95	NAD(P)-binding domain protein
GO:0016491	4	7.11E-03	23633221	MAA_11773	-1.72	hypothetical protein MAA_11773
GO:0016491	4	7.11E-03	19259418	MAA_05132	-1.66	NAD(P)-binding protein
GO:0016491	4	7.11E-03	19261793	MAA_07507	-1.59	Pyoverdine biosynthesis
GO:0016491	4	7.11E-03	19254364	MAA_00078	-1.39	FAD dependent monooxygenase
GO:0016491	4	7.11E-03	19263848	MAA_09562	-1.18	fatty acid hydroxylase superfamily protein
GO:0016491	4	7.11E-03	19262192	MAA_07906	-1.15	Alpha-hydroxy acid dehydrogenase, FMN-dependent
GO:0016491	4	7.11E-03	23632270	MAA_10821	-1.03	ubiquitin-conjugating enzyme
GO:0016491	4	7.11E-03	19262673	MAA_08387	1.06	short-chain dehydrogenase
GO:0016491	4	7.11E-03	19259242	MAA_04956	1.07	Amine oxidase

GO:0016491	4	7.11E-03	19263497	MAA_09211	1.19	hypothetical protein MAA_09211
GO:0016491	4	7.11E-03	19256546	MAA_02260	1.62	sarcosine oxidase
GO:0016491	4	7.11E-03	19255639	MAA_01353	1.69	Oxoglutarate/iron-dependent oxygenase
GO:0016491	4	7.11E-03	19258293	MAA_04007	1.95	alcohol dehydrogenase GroES domain-containing protein
GO:0016491	4	7.11E-03	19262218	MAA_07932	2.17	Pyruvate/2-oxoglutarate dehydrogenase
GO:0016491	4	7.11E-03	19264319	MAA_10033	2.28	polyketide synthase
GO:0016491	4	7.11E-03	19260992	MAA_06706	2.30	short-chain dehydrogenase
GO:0016491	4	7.11E-03	19264505	MAA_10219	2.44	FAD/FMN-containing isoamyl alcohol oxidase MreA
GO:0009395	8	7.16E-03	19259306	MAA_05020	-1.25	phospholipase-like protein
GO:0009395	8	7.16E-03	19260910	MAA_06624	-1.11	lysophospholipase precursor
GO:0016708	6	7.16E-03	19261778	MAA_07492	-1.78	c6 zinc finger domain containing protein
GO:0016708	6	7.16E-03	23632897	MAA_11449	-2.96	Ankyrin repeat-containing domain protein
GO:0031638	9	7.16E-03	19260495	MAA_06209	-3.07	Concanavalin A-like lectin/glucanase
GO:0031638	9	7.16E-03	19263490	MAA_09204	-5.66	Concanavalin A-like lectin/glucanase
GO:0046148	5	8.20E-03	19257525	MAA_03239	-2.33	Beta-ketoacyl synthase
GO:0046148	5	8.20E-03	19257527	MAA_03241	-1.82	glutamyl-tRNA(Gln) amidotransferase
GO:0046148	5	8.20E-03	23632196	MAA_10747	1.39	ankyrin repeat protein
GO:0010521	6	8.22E-03	23633205	MAA_11757	1.07	hypothetical protein MAA_11757
GO:0010521	6	8.22E-03	19263390	MAA_09104	1.10	aaa ATPase
GO:0010521	6	8.22E-03	19264434	MAA_10148	1.13	ATP-dependent DNA helicase PIF1
GO:0010521	6	8.22E-03	19264635	MAA_10349	1.28	ATP-dependent DNA helicase PIF1
GO:0010521	6	8.22E-03	19254648	MAA_00362	1.35	PIF1 protein
GO:0005657	6	9.69E-03	23633205	MAA_11757	1.07	hypothetical protein MAA_11757
GO:0005657	6	9.69E-03	19263390	MAA_09104	1.10	aaa ATPase
GO:0005657	6	9.69E-03	19264434	MAA_10148	1.13	ATP-dependent DNA helicase PIF1
GO:0005657	6	9.69E-03	19264635	MAA_10349	1.28	ATP-dependent DNA helicase PIF1
GO:0005657	6	9.69E-03	19254648	MAA_00362	1.35	PIF1 protein
GO:0030163	7	9.69E-03	19263004	MAA_08718	-6.63	Peptidase S8, subtilisin, Asp-active site protein
GO:0030163	7	9.69E-03	19255716	MAA_01430	-2.98	Peptidase A1
GO:0030163	7	9.69E-03	19262512	MAA_08226	-2.72	Peptidase aspartic, active site protein
GO:0030163	7	9.69E-03	23632201	MAA_10752	-1.74	triacylglycerol lipase-like protein
GO:0030163	7	9.69E-03	19255800	MAA_01514	-1.18	Peptidase A1
GO:0043141	12	1.13E-02	19254648	MAA_00362	1.35	PIF1 protein
GO:0043141	12	1.13E-02	19263390	MAA_09104	1.10	aaa ATPase
GO:0043141	12	1.13E-02	19264434	MAA_10148	1.13	ATP-dependent DNA helicase PIF1
GO:0043141	12	1.13E-02	19264635	MAA_10349	1.28	ATP-dependent DNA helicase PIF1
GO:0043141	12	1.13E-02	23633205	MAA_11757	1.07	hypothetical protein MAA_11757
GO:1900563	6	1.29E-02	19254364	MAA_00078	-1.39	FAD dependent monooxygenase
GO:1900563	6	1.29E-02	19262652	MAA_08366	1.80	methyltransferase [Metarhizium robertsii]
GO:1900563	6	1.29E-02	19262904	MAA_08618	1.81	Phytanoyl-CoA dioxygenase PhyH
GO:1900563	6	1.29E-02	19262909	MAA_08623	2.21	phytanoyl-CoA dioxygenase
GO:1900560	6	1.29E-02	19254364	MAA_00078	-1.39	FAD dependent monooxygenase
GO:1900560	6	1.29E-02	19262652	MAA_08366	1.80	methyltransferase [Metarhizium robertsii]
GO:1900560	6	1.29E-02	19262904	MAA_08618	1.81	Phytanoyl-CoA dioxygenase PhyH
GO:1900560	6	1.29E-02	19262909	MAA_08623	2.21	phytanoyl-CoA dioxygenase
GO:0019439	6	1.39E-02	19261778	MAA_07492	-1.78	c6 zinc finger domain containing protein
GO:0019439	6	1.39E-02	23632897	MAA_11449	-2.96	Ankyrin repeat-containing domain protein
GO:0060003	12	1.39E-02	19264416	MAA_10130	-1.09	Major facilitator superfamily domain, general substrate transporter
GO:0060003	12	1.39E-02	23632275	MAA_10826	-1.04	meiotic recombination protein DMC1
GO:0005618	5	1.39E-02	19264559	MAA_10273	-4.71	Asparaginase/glutaminase
GO:0005618	5	1.39E-02	19259838	MAA_05552	1.24	ribosomal protein L1
GO:0008812	6	1.39E-02	19254343	MAA_00057	-1.53	choline dehydrogenase
GO:0008812	6	1.39E-02	19255901	MAA_01615	-1.48	glucose oxidase
GO:0032508	9	1.51E-02	23633205	MAA_11757	1.07	hypothetical protein MAA_11757
GO:0032508	9	1.51E-02	19263390	MAA_09104	1.10	aaa ATPase
GO:0032508	9	1.51E-02	19264434	MAA_10148	1.13	ATP-dependent DNA helicase PIF1
GO:0032508	9	1.51E-02	19264635	MAA_10349	1.28	ATP-dependent DNA helicase PIF1
GO:0032508	9	1.51E-02	19254648	MAA_00362	1.35	PIF1 protein
GO:0006260	8	1.73E-02	23633205	MAA_11757	1.07	hypothetical protein MAA_11757
GO:0006260	8	1.73E-02	19263390	MAA_09104	1.10	aaa ATPase
GO:0006260	8	1.73E-02	19264434	MAA_10148	1.13	ATP-dependent DNA helicase PIF1

GO:0006260	8	1.73E-02	19264635	MAA_10349	1.28	ATP-dependent DNA helicase PIF1
GO:0006260	8	1.73E-02	19254648	MAA_00362	1.35	PIF1 protein
GO:0045461	7	1.87E-02	19260861	MAA_06575	-4.95	Beta-ketoacyl synthase
GO:0045461	7	1.87E-02	19264708	MAA_10422	-3.24	hypothetical protein MAA_10422
GO:0045461	7	1.87E-02	19264330	MAA_10044	-1.78	Cytochrome P450, DtxS2
GO:0045461	7	1.87E-02	19263397	MAA_09111	1.01	NUDIX hydrolase domain protein
GO:0045461	7	1.87E-02	19262652	MAA_08366	1.80	methyltransferase [Metarhizium robertsii]
GO:0045461	7	1.87E-02	19264488	MAA_10202	14.39	Beta-ketoacyl synthase
GO:0001765	7	2.23E-02	19264416	MAA_10130	-1.09	Major facilitator superfamily domain, general substrate transporter
GO:0001765	7	2.23E-02	23632275	MAA_10826	-1.04	meiotic recombination protein DMC1
GO:1900787	7	2.23E-02	19257525	MAA_03239	-2.33	Beta-ketoacyl synthase
GO:1900787	7	2.23E-02	23632196	MAA_10747	1.39	ankyrin repeat protein
GO:0001848	5	2.23E-02	19257525	MAA_03239	-2.33	Beta-ketoacyl synthase
GO:0001848	5	2.23E-02	23632196	MAA_10747	1.39	ankyrin repeat protein
GO:1900793	7	2.23E-02	19255257	MAA_00971	-4.81	Aromatic prenyltransferase, DMATS type
GO:1900793	7	2.23E-02	19260861	MAA_06575	-4.95	Beta-ketoacyl synthase
GO:0015074	8	2.41E-02	19255677	MAA_01391	1.06	transposase
GO:0015074	8	2.41E-02	19264758	MAA_10472	1.26	gag-pol polyprotein
GO:0015074	8	2.41E-02	23632364	MAA_10915	1.41	hypothetical protein MAA_10915
GO:0000002	7	2.52E-02	19254648	MAA_00362	1.35	PIF1 protein
GO:0000002	7	2.52E-02	19263390	MAA_09104	1.10	aaa ATPase
GO:0000002	7	2.52E-02	19264434	MAA_10148	1.13	ATP-dependent DNA helicase PIF1
GO:0000002	7	2.52E-02	19264635	MAA_10349	1.28	ATP-dependent DNA helicase PIF1
GO:0000002	7	2.52E-02	23633205	MAA_11757	1.07	hypothetical protein MAA_11757
GO:0006810	5	2.82E-02	19263527	MAA_09241	-2.28	cysteine-rich secretory protein
GO:0006810	5	2.82E-02	19262202	MAA_07916	-2.26	major facilitator superfamily transporter
GO:0006810	5	2.82E-02	19263703	MAA_09417	-1.30	oligopeptide transporter
GO:0006810	5	2.82E-02	19263316	MAA_09030	1.02	Sodium/solute symporter
GO:0006810	5	2.82E-02	19258027	MAA_03741	1.57	Major facilitator superfamily domain, general substrate transporter
GO:0009116	8	3.14E-02	19262115	MAA_07829	-4.38	WD40 repeat-like-containing domain protein
GO:0009116	8	3.14E-02	19263074	MAA_08788	-3.60	ankyrin repeat-containing protein
GO:0009116	8	3.14E-02	19264518	MAA_10232	-1.88	ankyrin repeat containing protein
GO:0009116	8	3.14E-02	19254338	MAA_00052	-1.71	Nucleoside phosphorylase domain protein
GO:0009116	8	3.14E-02	19255698	MAA_01412	-1.06	Ankyrin repeat-containing domain protein
GO:0003700	4	3.18E-02	23633117	MAA_11669	-13.77	hypothetical protein MAA_11669
GO:0003700	4	3.18E-02	19255215	MAA_00929	-2.17	Ribonuclease H-like protein
GO:0003700	4	3.18E-02	19257739	MAA_03453	-1.21	fungal specific transcription factor
GO:0003700	4	3.18E-02	23632363	MAA_10914	1.00	pol-like protein
GO:0003700	4	3.18E-02	19262889	MAA_08603	1.04	transcription factor Cys6
GO:0003700	4	3.18E-02	23633175	MAA_11727	1.08	FluG domain-containing protein
GO:0003700	4	3.18E-02	19254667	MAA_00381	1.11	Ribonuclease H-like protein
GO:0003700	4	3.18E-02	19263392	MAA_09106	1.12	FluG domain-containing protein
GO:0003700	4	3.18E-02	19264446	MAA_10160	1.20	transposase-like protein
GO:0003700	4	3.18E-02	19264853	MAA_10567	1.42	restless-like transposase
GO:1902181	7	3.24E-02	19262904	MAA_08618	1.81	Phytanoyl-CoA dioxygenase PhyH
GO:1902181	7	3.24E-02	19262909	MAA_08623	2.21	phytanoyl-CoA dioxygenase
GO:0018101	11	3.24E-02	19254780	MAA_00494	1.36	protein-arginine deiminase type-4
GO:0018101	11	3.24E-02	19262135	MAA_07849	-3.25	protein-arginine deiminase type-4
GO:0043324	6	3.24E-02	19257525	MAA_03239	-2.33	Beta-ketoacyl synthase
GO:0043324	6	3.24E-02	23632196	MAA_10747	1.39	ankyrin repeat protein
GO:0042710	5	3.24E-02	19264416	MAA_10130	-1.09	Major facilitator superfamily domain, general substrate transporter
GO:0042710	5	3.24E-02	23632275	MAA_10826	-1.04	meiotic recombination protein DMC1
GO:0071577	11	3.24E-02	19260674	MAA_06388	-1.90	Cd2+/Zn2+ transporter protein
GO:0071577	11	3.24E-02	19264673	MAA_10387	-1.89	ZIP Zinc transporter
GO:0009058	4	3.33E-02	19258721	MAA_04435	-1.23	Salicylate synthase
GO:0009058	4	3.33E-02	19261231	MAA_06945	-1.05	glycosyltransferase family 4 protein
GO:0009058	4	3.33E-02	19262903	MAA_08617	1.87	aromatic amino acid aminotransferase
GO:0009058	4	3.33E-02	19263977	MAA_09691	1.17	1-aminocyclopropane-1-carboxylate synthase
GO:0050660	6	3.34E-02	19263838	MAA_09552	-3.11	FAD-binding, type 2
GO:0050660	6	3.34E-02	19259153	MAA_04867	-2.08	Pyridine nucleotide-disulfide oxidoreductase
GO:0050660	6	3.34E-02	19254343	MAA_00057	-1.53	choline dehydrogenase

GO:0050660	6	3.34E-02	19255901	MAA_01615	-1.48	glucose oxidase
GO:0050660	6	3.34E-02	23632270	MAA_10821	-1.03	ubiquitin-conjugating enzyme
GO:0050660	6	3.34E-02	19262218	MAA_07932	2.17	Pyruvate/2-oxoglutarate dehydrogenase
GO:0050660	6	3.34E-02	19264505	MAA_10219	2.44	FAD/FMN-containing isoamyl alcohol oxidase MreA
GO:0016709	6	4.22E-02	19255798	MAA_01512	-2.15	Cytochrome P450 CYP5148B3
GO:0016709	6	4.22E-02	19264499	MAA_10213	-1.34	Cytochrome P450 CYP5317A1
GO:0016709	6	4.22E-02	19255582	MAA_01296	1.94	Cytochrome P450 CYP620N1
GO:1900584	6	4.22E-02	19260861	MAA_06575	-4.95	Beta-ketoacyl synthase
GO:1900584	6	4.22E-02	19262652	MAA_08366	1.80	methyltransferase [Metarhizium robertsii]
GO:1900584	6	4.22E-02	19264488	MAA_10202	14.39	Beta-ketoacyl synthase
GO:0022857	4	4.31E-02	19263051	MAA_08765	-1.78	sugar transporter-like protein
GO:0022857	4	4.31E-02	19264281	MAA_09995	-1.63	major facilitator superfamily transporter
GO:0022857	4	4.31E-02	19256624	MAA_02338	-1.55	General substrate transporter
GO:0022857	4	4.31E-02	19256697	MAA_02411	-1.06	alpha glucoside transporter
GO:0022857	4	4.31E-02	19256607	MAA_02321	2.02	Major facilitator superfamily domain, general substrate transporter
GO:0022857	4	4.31E-02	19257555	MAA_03269	5.05	membrane transporter
GO:0051537	6	4.39E-02	19261778	MAA_07492	-1.78	c6 zinc finger domain containing protein
GO:0051537	6	4.39E-02	23632897	MAA_11449	-2.96	Ankyrin repeat-containing domain protein
GO:0006066	5	4.39E-02	19254343	MAA_00057	-1.53	choline dehydrogenase
GO:0006066	5	4.39E-02	19255901	MAA_01615	-1.48	glucose oxidase
GO:0045121	6	4.39E-02	19264416	MAA_10130	-1.09	Major facilitator superfamily domain, general substrate transporter
GO:0045121	6	4.39E-02	23632275	MAA_10826	-1.04	meiotic recombination protein DMC1
GO:0042243	5	4.39E-02	19257525	MAA_03239	-2.33	Beta-ketoacyl synthase
GO:0042243	5	4.39E-02	23632196	MAA_10747	1.39	ankyrin repeat protein
GO:0071931	10	4.97E-02	19264559	MAA_10273	-4.71	Asparaginase/glutaminase
GO:0019500	7	4.97E-02	19264611	MAA_10325	1.69	Nitrilase/cyanide hydratase and apolipoprotein N-acyltransferase
GO:0032088	7	4.97E-02	23632752	MAA_11304	-1.11	maleylacetate reductase
GO:0045148	9	4.97E-02	19262821	MAA_08535	11.37	tripeptidyl peptidase a
GO:0017064	7	4.97E-02	19265002	MAA_10718	1.64	hypothetical protein MAA_10718
GO:0051092	7	4.97E-02	23632752	MAA_11304	-1.11	maleylacetate reductase
GO:0032736	8	4.97E-02	23632752	MAA_11304	-1.11	maleylacetate reductase
GO:0035229	8	4.97E-02	19259926	MAA_05640	-1.01	aldo-keto reductase family protein
GO:0050701	12	4.97E-02	23632752	MAA_11304	-1.11	maleylacetate reductase
GO:2000100	8	4.97E-02	19264830	MAA_10544	1.01	Protein kinase, ATP binding site
GO:0031579	6	4.97E-02	19263663	MAA_09377	-2.01	SPFH domain/Band 7 family protein
GO:0048288	9	4.97E-02	19256359	MAA_02073	1.21	nuclear membrane fusion protein Kar5
GO:1900196	8	4.97E-02	19260324	MAA_06038	-1.57	pH regulatory protein mrPacC
GO:0046565	7	4.97E-02	19264141	MAA_09855	1.22	4-hydroxyphenylpyruvate dioxygenase
GO:0015189	7	4.97E-02	19261806	MAA_07520	1.00	major facilitator superfamily transporter
GO:0019619	9	4.97E-02	19256410	MAA_02124	-1.05	menaquinone methyltransferase related to dlpA protein
GO:0032753	8	4.97E-02	23632752	MAA_11304	-1.11	maleylacetate reductase
GO:0019566	8	4.97E-02	19254294	MAA_00008	-1.27	Alpha-L-arabinofuranosidase B, catalytic
GO:1900602	6	4.97E-02	19260861	MAA_06575	-4.95	Beta-ketoacyl synthase
GO:0050537	7	4.97E-02	19262832	MAA_08546	1.46	Amidase
GO:0045111	7	4.97E-02	19262135	MAA_07849	-3.25	protein-arginine deiminase type-4
GO:0051607	5	4.97E-02	23632752	MAA_11304	-1.11	maleylacetate reductase
GO:0006954	6	4.97E-02	23632752	MAA_11304	-1.11	maleylacetate reductase
GO:0072741	7	4.97E-02	19264830	MAA_10544	1.01	Protein kinase, ATP binding site
GO:1900695	7	4.97E-02	19260324	MAA_06038	-1.57	pH regulatory protein mrPacC
GO:1903714	12	4.97E-02	19261806	MAA_07520	1.00	major facilitator superfamily transporter
GO:0006530	12	4.97E-02	19264559	MAA_10273	-4.71	Asparaginase/glutaminase
GO:0017109	6	4.97E-02	19259926	MAA_05640	-1.01	aldo-keto reductase family protein
GO:0050713	10	4.97E-02	23632752	MAA_11304	-1.11	maleylacetate reductase
GO:0032754	8	4.97E-02	23632752	MAA_11304	-1.11	maleylacetate reductase
GO:0000257	7	4.97E-02	19264611	MAA_10325	1.69	Nitrilase/cyanide hydratase and apolipoprotein N-acyltransferase
GO:0000721	7	4.97E-02	19263692	MAA_09406	1.17	hypothetical protein MAA_09406
GO:2000553	12	4.97E-02	23632752	MAA_11304	-1.11	maleylacetate reductase
GO:0008689	8	4.97E-02	19260313	MAA_06027	1.92	type 11 methyltransferase
GO:0002674	10	4.97E-02	23632752	MAA_11304	-1.11	maleylacetate reductase
GO:0044546	8	4.97E-02	23632752	MAA_11304	-1.11	maleylacetate reductase
GO:0051560	12	4.97E-02	19259729	MAA_05443	-1.96	siderophore biosynthesis protein

GO:0033934	7	4.97E-02	19263052	MAA_08766	-1.67	maltase
GO:0050421	7	4.97E-02	19263202	MAA_08916	1.45	multicopper oxidase
GO:0000907	7	4.97E-02	19263874	MAA_09588	1.89	TfdA family Taurine catabolism dioxygenase TauD
GO:0044273	6	4.97E-02	19263874	MAA_09588	1.89	TfdA family Taurine catabolism dioxygenase TauD
GO:0071222	8	4.97E-02	23632752	MAA_11304	-1.11	maleylacetate reductase
GO:0036444	13	4.97E-02	19259729	MAA_05443	-1.96	siderophore biosynthesis protein
GO:0030600	7	4.97E-02	19257778	MAA_03492	1.14	feruloyl esterase B
GO:2000321	9	4.97E-02	23632752	MAA_11304	-1.11	maleylacetate reductase
GO:0003858	7	4.97E-02	19262824	MAA_08538	1.13	3-hydroxyacid dehydrogenase/reductase
GO:0072559	5	4.97E-02	23632752	MAA_11304	-1.11	maleylacetate reductase
GO:0032621	5	4.97E-02	23632752	MAA_11304	-1.11	maleylacetate reductase
GO:0045630	9	4.97E-02	23632752	MAA_11304	-1.11	maleylacetate reductase
GO:1900813	7	4.97E-02	19260861	MAA_06575	-4.95	Beta-ketoacyl synthase
GO:0047443	7	4.97E-02	19256410	MAA_02124	-1.05	menaquinone methyltransferase related to dlpA protein
GO:0050718	10	4.97E-02	23632752	MAA_11304	-1.11	maleylacetate reductase
GO:0004084	7	4.97E-02	19264325	MAA_10039	1.44	subgroup IIIi aminotransferase
GO:0046556	7	4.97E-02	19254294	MAA_00008	-1.27	Alpha-L-arabinofuranosidase B, catalytic
GO:0004395	9	4.97E-02	19260313	MAA_06027	1.92	type 11 methyltransferase
GO:0004574	9	4.97E-02	19263052	MAA_08766	-1.67	maltase
GO:0034079	10	4.97E-02	19263692	MAA_09406	1.17	hypothetical protein MAA_09406
GO:0008948	7	4.97E-02	19256410	MAA_02124	-1.05	menaquinone methyltransferase related to dlpA protein
GO:0044595	9	4.97E-02	19260313	MAA_06027	1.92	type 11 methyltransferase
GO:0006877	12	4.97E-02	19260674	MAA_06388	-1.90	Cd2+/Zn2+ transporter protein
GO:0007028	5	4.97E-02	19262135	MAA_07849	-3.25	protein-arginine deiminase type-4
GO:0006919	11	4.97E-02	23632752	MAA_11304	-1.11	maleylacetate reductase
GO:0032611	6	4.97E-02	23632752	MAA_11304	-1.11	maleylacetate reductase
GO:1902475	12	4.97E-02	19261806	MAA_07520	1.00	major facilitator superfamily transporter
GO:0030196	7	4.97E-02	19264611	MAA_10325	1.69	Nitrilase/cyanide hydratase and apolipoprotein N-acyltransferase
GO:0007010	6	4.97E-02	19262135	MAA_07849	-3.25	protein-arginine deiminase type-4
GO:0030907	7	4.97E-02	19264559	MAA_10273	-4.71	Asparaginase/glutaminase
GO:0004067	7	4.97E-02	19264559	MAA_10273	-4.71	Asparaginase/glutaminase
GO:0015182	8	4.97E-02	19261806	MAA_07520	1.00	major facilitator superfamily transporter
GO:0071947	10	4.97E-02	19262933	MAA_08647	-1.04	hypothetical protein MAA_08647
GO:0016139	8	4.97E-02	19263916	MAA_09630	-2.03	alpha-N-acetylgalactosaminidase
GO:1990246	9	4.97E-02	19259729	MAA_05443	-1.96	siderophore biosynthesis protein
GO:0015067	6	4.97E-02	19264062	MAA_09776	1.15	lysine amidinotransferase
GO:0008425	9	4.97E-02	19260313	MAA_06027	1.92	type 11 methyltransferase
GO:0043143	10	4.97E-02	19262135	MAA_07849	-3.25	protein-arginine deiminase type-4
GO:0044596	8	4.97E-02	19260313	MAA_06027	1.92	type 11 methyltransferase
GO:0036151	10	4.97E-02	19259306	MAA_05020	-1.25	phospholipase-like protein
GO:1990591	13	4.97E-02	19261806	MAA_07520	1.00	major facilitator superfamily transporter
GO:0015292	7	4.97E-02	19259729	MAA_05443	-1.96	siderophore biosynthesis protein
GO:1901482	10	4.97E-02	19261806	MAA_07520	1.00	major facilitator superfamily transporter
GO:0005668	8	4.97E-02	19255392	MAA_01106	1.07	Ubiquitin supergroup
GO:1900549	5	4.97E-02	19260324	MAA_06038	-1.57	pH regulatory protein mrPacC

Table S4. Summation of GO function classes and terms enriched to *M. robertsii* Δ*cfp1* versus WT strain.

GO_function class / GO_ term enriched	Counts of genes		
	down	up	subtotal
**Cellular Component**			
Cellular component	147	106	253
Extracellular region	16	1	17
Extracellular space	4	1	5
Replication fork	0	5	5
Cell	1	1	2
Cell wall	1	1	2
Membrane raft	2	0	2
Intermediate filament cytoskeleton	1	0	1
Glutamate-cysteine ligase complex	1	0	1
NLRP3 inflammasome complex	1	0	1
MBF transcription complex	1	0	1
Uniplex complex	1	0	1
RNA polymerase transcription factor SL1 complex	0	1	1
**Biological Process**			
Oxidation-reduction process	17	15	32
Proteolysis	8	6	14
Regulation of transcription by RNA polymerase II	4	6	10
Secondary metabolic process	7	3	10
Negative regulation of telomerase activity	0	10	10
Monodictyphenone biosynthetic process	3	3	6
Sterigmatocystin biosynthetic process	3	3	6
Melanin biosynthetic process	4	1	5
G-quadruplex DNA unwinding	0	5	5
Protein catabolic process	5	0	5
DNA duplex unwinding	0	5	5
DNA replication	0	5	5
Mitochondrial genome maintenance	0	5	5
Transport	3	2	5
Nucleoside metabolic process	5	0	5
Dehydroaustinol biosynthetic process	1	3	4
Austinol biosynthetic process	1	3	4
Biosynthetic process	2	2	4
Xanthone-containing compound biosynthetic process	3	0	3
Pigment biosynthetic process	2	1	3
Austinol biosynthetic process	3	0	3
DNA integration	0	3	3
O-orsellinic acid biosynthetic process	1	2	3
Positive regulation of NF-kappaB transcription factor activity	3	0	3
Phospholipid catabolic process	2	0	2
Zymogen activation	2	0	2
Copper ion export	2	0	2
Membrane raft assembly	2	0	2
Naphtho-gamma-pyrone biosynthetic process	1	1	2
Shamixanthone biosynthetic process	2	0	2
Verruculogen biosynthetic process	0	2	2
Protein citrullination	1	1	2
Pigment metabolic process involved in developmental pigmentation	1	1	2
Biofilm formation	2	0	2
Zinc II ion transmembrane transport	2	0	2
Alcohol metabolic process	2	0	2
Asexual spore wall assembly	1	1	2
Positive regulation of transcription involved in G1/S transition of mitotic cell cycle	1	0	1
Cyanide catabolic process	0	1	1
Negative regulation of NF-kappaB transcription factor activity	1	0	1
Interleukin-1 secretion	1	0	1
Regulation of establishment or maintenance of bipolar cell polarity regulating cell shape	0	1	1

Membrane raft organization	1		1
Nuclear membrane fusion involved in karyogamy	0	1	1
Regulation of penicillin biosynthetic process	1	0	1
3,4-dihydroxybenzoate catabolic process	1	0	1
Positive regulation of interleukin-4 production	1	0	1
Arabinose metabolic process	1	0	1
Endocrocin biosynthetic process	1	0	1
Defense response to virus	1	0	1
Inflammatory response	1	0	1
Protein localization to cell division site	0	1	1
Regulation of N',N'',N'''-triacetylfusarinine C biosynthetic process	1	0	1
Isoleucine transmembrane transport	0	1	1
Asparagine catabolic process	1	0	1
Negative regulation of interleukin-1 beta secretion	1	0	1
Positive regulation of interleukin-5 production	1	0	1
Positive regulation of T-helper 2 cell cytokine production	1	0	1
Negative regulation of acute inflammatory response	1	0	1
NLRP3 inflammasome complex assembly	1	0	1
Mitochondrial calcium ion homeostasis	1	0	1
Sulfur compound catabolic process	0	1	1
Cellular response to lipopolysaccharide	1	0	1
Calcium import into the mitochondrion	1	0	1
Positive regulation of T-helper 17 cell differentiation	0	1	1
Interleukin-18 production	1	0	1
Positive regulation of T-helper 2 cell differentiation	1	0	1
Monodictyphenone metabolic process	1	0	1
Positive regulation of interleukin-1 beta secretion	1	0	1
Butanediol biosynthetic process	0	1	1
Cellular cobalt ion homeostasis	1	0	1
Cytoplasm organization	1	0	1
Activation of cysteine-type endopeptidase activity involved in apoptotic process	1	0	1
Interleukin-1 beta production	1	0	1
L-alpha-amino acid transmembrane transport	0	1	1
Cytoskeleton organization	1	0	1
Protein deubiquitination involved in ubiquitin-dependent protein catabolic process	1	0	1
Glycoside catabolic process	1	0	1
Regulation of translation by machinery localization	1	0	1
Phosphatidylcholine acyl-chain remodeling	1	0	1
Asparagine transmembrane import into vacuole	0	1	1
L-lysine import into vacuole involved in cellular response to nitrogen starvation	0	1	1
N',N'',N'''-triacetylfusarinine C metabolic process	1	0	1
**Molecular Function**			
Catalytic activity	13	13	26
Oxidoreductase activity	13	10	23
DNA binding transcription factor activity	3	10	13
Serine-type endopeptidase activity	9	3	12
RNA polymerase II regulatory region sequence-specific DNA binding	2	6	8
Phosphopantetheine binding	6	2	8
Transferase activity, transferring acyl groups other than amino-acyl groups	4	4	8
Transferase activity	3	5	8
Flavin adenine dinucleotide binding	5	2	7
Aspartic-type endopeptidase activity	6	0	6
Transmembrane transporter activity	4	2	6
Dioxygenase activity	3	2	5
Oxidoreductase activity, acting on CH-OH group of donors	4	1	5
Telomerase inhibitor activity	0	5	5
ATP-dependent 5'-3' DNA helicase activity	0	5	5
Cation-transporting ATPase activity	4	0	4
Protein-arginine deiminase activity	2	2	4
Serine-type peptidase activity	1	3	4
Oxidoreductase activity, acting on paired donors, with ... one atom of oxygen	2	1	3

Oxidoreductase activity, acting on paired donors, with ... two atoms of oxygen	2	0	2
Choline dehydrogenase activity	2	0	2
Complement binding	1	1	2
2 iron, 2 sulfur cluster binding	2	0	2
Tripeptide aminopeptidase activity	0	1	1
Fatty acid amide hydrolase activity	0	1	1
3-dehydroshikimate dehydratase activity	0	1	1
L-lysine transmembrane transporter activity	0	1	1
Mandelamide amidase activity	0	1	1
Nitrilase activity	0	1	1
(R,R)-butanediol dehydrogenase activity	0	1	1
3-demethylubiquinone-9 3-O-methyltransferase activity	0	1	1
Glucan 1,4-alpha-maltotriohydrolase activity	1	0	1
Nitrite reductase (NO-forming) activity	0	1	1
Sulfonate dioxygenase activity	0	1	1
Feruloyl esterase activity	1	0	1
3-hydroxybutyrate dehydrogenase activity	1	0	1
4-hydroxy-4-methyl-2-oxoglutarate aldolase activity	1	0	1
Branched-chain-amino-acid transaminase activity	0	1	1
Alpha-L-arabinofuranosidase activity	1	0	1
Hexaprenyldihydroxybenzoate methyltransferase activity	0	1	1
Oligo-1,6-glucosidase activity	1	0	1
Oxaloacetate decarboxylase activity	1	0	1
Decaprenyldihydroxybenzoate methyltransferase activity	0	1	1
Cyanide hydratase activity	0	1	1
Asparaginase activity	1	0	1
L-asparagine transmembrane transporter activity	0	1	1
Amidinotransferase activity	0	1	1
2-polyprenyl-6-methoxy-1,4-benzoquinone methyltransferase activity	0	1	1
3-demethylubiquinone-10 3-O-methyltransferase activity	0	1	1
Uniporter activity	1	0	1
**Total:**	**406**	**311**	**717**

Table S5. KEGG pathway analysis of differential expressed genes in *M. robertsii* Δ*cfp1* versus WT strain.

Pathway_id	Pathway_name	*p*-value	KO_entry	EC	Gene_ID	Gene_name	$\log_2 R$	Description
ko00260	Glycine, serine and threonine metabolism	6.27E-04	K00108	EC:1.1.99.1	19254343	MAA_00057	-1.53	choline dehydrogenase
ko00260	Glycine, serine and threonine metabolism	6.27E-04	K00108	EC:1.1.99.1	19255901	MAA_01615	-1.48	glucose oxidase
ko00260	Glycine, serine and threonine metabolism	6.27E-04	K00306	EC:1.5.3.1;EC:1.5.3.7	19257557	MAA_03271	-1.47	flavin-binding monooxygenase
ko00260	Glycine, serine and threonine metabolism	6.27E-04	K00865	EC:2.7.1.165	19262758	MAA_08472	-1.32	Glycerate kinase
ko00260	Glycine, serine and threonine metabolism	6.27E-04	K00108	EC:1.1.99.1	23632203	MAA_10754	-1.15	gag-pol polyprotein
ko00260	Glycine, serine and threonine metabolism	6.27E-04	K00600	EC:2.1.2.1	19262129	MAA_07843	-1.02	patatin-like phospholipase
ko00260	Glycine, serine and threonine metabolism	6.27E-04	K00613	EC:2.1.4.1	19264062	MAA_09776	1.15	lysine amidinotransferase
ko00260	Glycine, serine and threonine metabolism	6.27E-04	K01733	EC:4.2.3.1	19255583	MAA_01297	1.38	haloacid dehalogenase
ko00260	Glycine, serine and threonine metabolism	6.27E-04	K00306	EC:1.5.3.1;EC:1.5.3.7	19256546	MAA_02260	1.62	sarcosine oxidase
ko00260	Glycine, serine and threonine metabolism	6.27E-04	K00831	EC:2.6.1.52	19262906	MAA_08620	1.84	nmrA-like family protein
ko00260	Glycine, serine and threonine metabolism	6.27E-04	K00108	EC:1.1.99.1	19262974	MAA_08688	2.00	CFEM domain protein
ko00260	Glycine, serine and threonine metabolism	6.27E-04	K00382	EC:1.8.1.4	19262218	MAA_07932	2.17	Pyruvate/2-oxoglutarate dehydrogenase
ko00061	Fatty acid biosynthesis	1.09E-03	K00059	EC:1.1.1.100	19257526	MAA_03240	-3.79	hypothetical protein MAA_03240
ko00061	Fatty acid biosynthesis	1.09E-03	K00059	EC:1.1.1.100	19264708	MAA_10422	-3.24	hypothetical protein MAA_10422
ko00061	Fatty acid biosynthesis	1.09E-03	K01897	EC:6.2.1.3	19263892	MAA_09606	-1.21	FAD binding domain-containing protein
ko00061	Fatty acid biosynthesis	1.09E-03	K01897	EC:6.2.1.3	23632270	MAA_10821	-1.03	ubiquitin-conjugating enzyme
ko00061	Fatty acid biosynthesis	1.09E-03	K01897	EC:6.2.1.3	19258184	MAA_03898	1.14	long-chain-fatty-acid-CoA ligase
ko00061	Fatty acid biosynthesis	1.09E-03	K00059	EC:1.1.1.100	19260992	MAA_06706	2.30	short-chain dehydrogenase
ko00061	Fatty acid biosynthesis	1.09E-03	K01897	EC:6.2.1.3	19264505	MAA_10219	2.44	FAD/FMN-containing isoamyl alcohol oxidase MreA
ko00564	Glycerophospholipid metabolism	2.01E-03	K01613	EC:4.1.1.65	19261596	MAA_07310	-1.34	integral membrane family protein
ko00564	Glycerophospholipid metabolism	2.01E-03	K13333	EC:3.1.1.5	19259306	MAA_05020	-1.25	phospholipase-like protein
ko00564	Glycerophospholipid metabolism	2.01E-03	K13333	EC:3.1.1.5	19260910	MAA_06624	-1.11	lysophospholipase precursor
ko00564	Glycerophospholipid metabolism	2.01E-03	K01049	EC:3.1.1.7	19262271	MAA_07985	-1.09	carboxylesterase/lipase domain protein
ko00564	Glycerophospholipid metabolism	2.01E-03	K18693	EC:3.1.3.81;EC:3.1.3.4	19256214	MAA_01928	-1.02	Ubiquitin 3 binding protein But2
ko00564	Glycerophospholipid metabolism	2.01E-03	K01613	EC:4.1.1.65	19264450	MAA_10164	1.14	reverse transcriptase, RNaseH
ko00564	Glycerophospholipid metabolism	2.01E-03	K13333	EC:3.1.1.5	19258326	MAA_04040	1.41	alpha/beta hydrolase
ko00564	Glycerophospholipid metabolism	2.01E-03	K06123	EC:1.1.1.101	19258327	MAA_04041	1.61	hypothetical protein MAA_04041
ko00564	Glycerophospholipid metabolism	2.01E-03	K06123	EC:1.1.1.101	23633070	MAA_11622	1.71	hypothetical protein MAA_11622
ko00564	Glycerophospholipid metabolism	2.01E-03	K01114	EC:3.1.4.3	19255954	MAA_01668	1.84	Phosphoesterase
ko00564	Glycerophospholipid metabolism	2.01E-03	K01613	EC:4.1.1.65	19260312	MAA_06026	2.08	phosphatidylserine decarboxylase family protein
ko00350	Tyrosine metabolism	6.88E-03	K00129	EC:1.2.1.5	19258281	MAA_03995	-1.51	ThiJ/PfpI family protein
ko00350	Tyrosine metabolism	6.88E-03	K00129	EC:1.2.1.5	19260050	MAA_05764	-1.31	ThiJ/PfpI
ko00350	Tyrosine metabolism	6.88E-03	K13953	EC:1.1.1.1	19256915	MAA_02629	1.05	Alcohol dehydrogenase superfamily, zinc-type
ko00350	Tyrosine metabolism	6.88E-03	K00450	EC:1.13.11.4	19256665	MAA_02379	1.12	Aromatic-ring hydroxylase-like protein
ko00350	Tyrosine metabolism	6.88E-03	K00129	EC:1.2.1.5	19259248	MAA_04962	1.12	aldehyde dehydrogenase
ko00350	Tyrosine metabolism	6.88E-03	K00457	EC:1.13.11.27	19264141	MAA_09855	1.22	4-hydroxyphenylpyruvate dioxygenase

ko00350	Tyrosine metabolism	6.88E-03	K00129	EC:1.2.1.5	19261170	MAA_06884	1.36	Aldehyde dehydrogenase
ko00350	Tyrosine metabolism	6.88E-03	K02510	EC:4.1.2.52	19260837	MAA_06551	1.39	macrophomate synthase
ko00350	Tyrosine metabolism	6.88E-03	K00838	EC:2.6.1.57;EC:2.6.1.39	19262903	MAA_08617	1.87	aromatic amino acid aminotransferase
ko00410	beta-Alanine metabolism	7.80E-03	K01580	EC:4.1.1.15	19264332	MAA_10046	-3.68	Aspartic acid decarboxylase, DtxS4
ko00410	beta-Alanine metabolism	7.80E-03	K00129	EC:1.2.1.5	19258281	MAA_03995	-1.51	ThiJ/PfpI family protein
ko00410	beta-Alanine metabolism	7.80E-03	K00129	EC:1.2.1.5	19260050	MAA_05764	-1.31	ThiJ/PfpI
ko00410	beta-Alanine metabolism	7.80E-03	K13366	EC:1.5.3.14;EC:1.5.3.16	19259242	MAA_04956	1.07	Amine oxidase
ko00410	beta-Alanine metabolism	7.80E-03	K00129	EC:1.2.1.5	19259248	MAA_04962	1.12	aldehyde dehydrogenase
ko00410	beta-Alanine metabolism	7.80E-03	K00129	EC:1.2.1.5	19261170	MAA_06884	1.36	Aldehyde dehydrogenase
ko00410	beta-Alanine metabolism	7.80E-03	K01580	EC:4.1.1.15	19264603	MAA_10317	1.98	pyridoxal-dependent decarboxylase domain protein
ko00360	Phenylalanine metabolism	1.09E-02	K00129	EC:1.2.1.5	19258281	MAA_03995	-1.51	ThiJ/PfpI family protein
ko00360	Phenylalanine metabolism	1.09E-02	K10437	EC:1.14.14.54	19264499	MAA_10213	-1.34	Cytochrome P450 CYP5317A1
ko00360	Phenylalanine metabolism	1.09E-02	K00129	EC:1.2.1.5	19260050	MAA_05764	-1.31	ThiJ/PfpI
ko00360	Phenylalanine metabolism	1.09E-02	K00129	EC:1.2.1.5	19259248	MAA_04962	1.12	aldehyde dehydrogenase
ko00360	Phenylalanine metabolism	1.09E-02	K00457	EC:1.13.11.27	19264141	MAA_09855	1.22	4-hydroxyphenylpyruvate dioxygenase
ko00360	Phenylalanine metabolism	1.09E-02	K00129	EC:1.2.1.5	19261170	MAA_06884	1.36	Aldehyde dehydrogenase
ko00360	Phenylalanine metabolism	1.09E-02	K01426	EC:3.5.1.4	19265002	MAA_10718	1.64	hypothetical protein MAA_10718
ko00360	Phenylalanine metabolism	1.09E-02	K00838	EC:2.6.1.57;EC:2.6.1.39	19262903	MAA_08617	1.87	aromatic amino acid aminotransferase
ko00052	Galactose metabolism	1.38E-02	K07407	EC:3.2.1.22	19263916	MAA_09630	-2.03	alpha-N-acetylgalactosaminidase
ko00052	Galactose metabolism	1.38E-02	K01182	EC:3.2.1.10	19263052	MAA_08766	-1.67	maltase
ko00052	Galactose metabolism	1.38E-02	K01784	EC:5.1.3.2	19261230	MAA_06944	-1.53	UDP-glucose 4-epimerase
ko00052	Galactose metabolism	1.38E-02	K00844	EC:2.7.1.1	19256497	MAA_02211	-1.24	hexokinase-1
ko00052	Galactose metabolism	1.38E-02	K01190	EC:3.2.1.23	19263915	MAA_09629	-1.08	Glycoside hydrolase, family 35
ko00340	Histidine metabolism	1.93E-02	K00129	EC:1.2.1.5	19258281	MAA_03995	-1.51	ThiJ/PfpI family protein
ko00340	Histidine metabolism	1.93E-02	K00129	EC:1.2.1.5	19260050	MAA_05764	-1.31	ThiJ/PfpI
ko00340	Histidine metabolism	1.93E-02	K00129	EC:1.2.1.5	19259248	MAA_04962	1.12	aldehyde dehydrogenase
ko00340	Histidine metabolism	1.93E-02	K20246	EC:2.1.1.44;EC:1.14.99.51	19259142	MAA_04856	1.23	DUF323 domain protein
ko00340	Histidine metabolism	1.93E-02	K00129	EC:1.2.1.5	19261170	MAA_06884	1.36	Aldehyde dehydrogenase
ko00750	Vitamin B6 metabolism	2.22E-02	K05275	EC:1.1.1.65	19264018	MAA_09732	1.02	Aldo/keto reductase
ko00750	Vitamin B6 metabolism	2.22E-02	K01733	EC:4.2.3.1	19255583	MAA_01297	1.38	haloacid dehalogenase
ko00750	Vitamin B6 metabolism	2.22E-02	K00831	EC:2.6.1.52	19262906	MAA_08620	1.84	nmrA-like family protein
ko04145	Phagosome	2.95E-02	K07374	NA	19264807	MAA_10521	-10.75	zinc knuckle
ko04145	Phagosome	2.95E-02	K02155	NA	19262115	MAA_07829	-4.38	WD40 repeat-like-containing domain protein
ko04145	Phagosome	2.95E-02	K02155	NA	19263074	MAA_08788	-3.60	ankyrin repeat-containing protein
ko04145	Phagosome	2.95E-02	K02147	NA	19264333	MAA_10047	-3.17	V-type ATPase
ko04145	Phagosome	2.95E-02	K02155	NA	19264518	MAA_10232	-1.88	ankyrin repeat containing protein
ko04145	Phagosome	2.95E-02	K00921	EC:2.7.1.150	19261146	MAA_06860	1.01	F-box domain protein
ko00460	Cyanoamino acid metabolism	3.39E-02	K01424	EC:3.5.1.1	19264559	MAA_10273	-4.71	Asparaginase/glutaminase
ko00460	Cyanoamino acid metabolism	3.39E-02	K01188	EC:3.2.1.21	19260432	MAA_06146	-1.06	glycoside hydrolase family 3 protein

ko00460	Cyanoamino acid metabolism	3.39E-02	K00600	EC:2.1.2.1	19262129	MAA_07843	-1.02	patatin-like phospholipase
ko00460	Cyanoamino acid metabolism	3.39E-02	K10675	EC:4.2.1.66	19264611	MAA_10325	1.69	Nitrilase/cyanide hydratase and apolipoprotein N-acyltransferase
ko00650	Butanoate metabolism	4.04E-02	K01580	EC:4.1.1.15	19264332	MAA_10046	-3.68	Aspartic acid decarboxylase, DtxS4
ko00650	Butanoate metabolism	4.04E-02	K00022	EC:1.1.1.35	19263128	MAA_08842	-1.45	NAD(P)-binding domain protein
ko00650	Butanoate metabolism	4.04E-02	K00004	EC:1.1.1.4;EC:1.1.1.-	19263692	MAA_09406	1.17	hypothetical protein MAA_09406
ko00650	Butanoate metabolism	4.04E-02	K00019	EC:1.1.1.30	19263497	MAA_09211	1.19	hypothetical protein MAA_09211
ko00650	Butanoate metabolism	4.04E-02	K01580	EC:4.1.1.15	19264603	MAA_10317	1.98	pyridoxal-dependent decarboxylase domain protein
ko00333	Prodigiosin biosyntheses	4.19E-02	K00059	EC:1.1.1.100	19257526	MAA_03240	-3.79	hypothetical protein MAA_03240
ko00333	Prodigiosin biosyntheses	4.19E-02	K00059	EC:1.1.1.100	19264708	MAA_10422	-3.24	hypothetical protein MAA_10422
ko00333	Prodigiosin biosyntheses	4.19E-02	K00059	EC:1.1.1.100	19260992	MAA_06706	2.30	short-chain dehydrogenase
ko00520	Amino sugar and nucleotide sugar metabolism	4.23E-02	K01183	EC:3.2.1.14	19263304	MAA_09018	-1.80	Glycoside hydrolase, subgroup, catalytic core
ko00520	Amino sugar and nucleotide sugar metabolism	4.23E-02	K01443	EC:3.5.1.25	19256493	MAA_02207	-1.71	carbohydrate esterase family 9 protein
ko00520	Amino sugar and nucleotide sugar metabolism	4.23E-02	K01784	EC:5.1.3.2	19261230	MAA_06944	-1.53	UDP-glucose 4-epimerase
ko00520	Amino sugar and nucleotide sugar metabolism	4.23E-02	K01183	EC:3.2.1.14	19256971	MAA_02685	-1.44	chitinase
ko00520	Amino sugar and nucleotide sugar metabolism	4.23E-02	K01183	EC:3.2.1.14	19262044	MAA_07758	-1.29	coagulation factor 5/8 type domain protein
ko00520	Amino sugar and nucleotide sugar metabolism	4.23E-02	K20844	EC:3.2.1.55	19254294	MAA_00008	-1.27	Alpha-L-arabinofuranosidase B, catalytic
ko00520	Amino sugar and nucleotide sugar metabolism	4.23E-02	K00844	EC:2.7.1.1	19256497	MAA_02211	-1.24	hexokinase-1
ko00520	Amino sugar and nucleotide sugar metabolism	4.23E-02	K01233	EC:3.2.1.132	19257096	MAA_02810	1.24	Fungal chitosanase

Table S6. Summation of KEGG pathways enriched to *M. robertsii* Δ*cfp1* versus WT strain.

KEGG pathway name	Counts of genes		
	Down	Up	Subtotal
Amino sugar and nucleotide sugar metabolism	7	1	8
Glycine, serine and threonine metabolism	6	6	12
Fatty acid biosynthesis	4	3	7
Glycerophospholipid metabolism	5	6	11
Tyrosine metabolism	2	7	9
beta-Alanine metabolism	3	4	7
Phenylalanine metabolism	3	5	8
Galactose metabolism	5	0	5
Histidine metabolism	2	3	5
Phagosome	5	1	6
Cyanoamino acid metabolism	3	1	4
Butanoate metabolism	2	3	5
Prodigiosin biosyntheses	2	1	3
Vitamin B6 metabolism	0	3	3
**Total:**	**49**	**44**	**93**

Table S7. Differentially expressed genes related to phenotypes and genome stability of *M. robertsii* Δ*cfp1* versus WT strain.

gene_id	gene_name	$\log_2 R$	*q*-value	Annotation
**Involved in cuticle degradation and virulence**				
19261241	MAA_06955	-11.71	1.04E-03	lipase 2
19263004	MAA_08718	-6.63	4.34E-10	Peptidase S8, subtilisin, Asp-active site protein
19259961	MAA_05675	-4.48	4.98E-44	alkaline serine protease P32
19255283	MAA_00997	-4.09	2.01E-12	peptidase family M28
23632468	MAA_11020	-3.77	2.95E-02	peptidase S1 domain protein
19256231	MAA_01945	-3.30	1.09E-25	fungalysin metallopeptidase
19263878	MAA_09592	-3.08	2.12E-25	Peptidase S1/S6, chymotrypsin/Hap
19255716	MAA_01430	-2.98	1.99E-13	Peptidase A1
19261160	MAA_06874	-2.59	2.30E-11	Necrosis inducing
19257548	MAA_03262	-2.40	3.01E-10	Peptidase S1/S6, chymotrypsin/Hap
19262601	MAA_08315	-2.30	1.80E-03	Glycoside hydrolase, chitinase active site protein
19263527	MAA_09241	-2.28	2.55E-02	cysteine-rich secretory protein
19254299	MAA_00013	-2.17	2.08E-04	lipase, class 3
19261774	MAA_07488	-2.11	3.47E-10	exo-1,3-beta-D-glucanase
19263304	MAA_09018	-1.80	1.84E-04	Glycoside hydrolase, subgroup, catalytic core
19262987	MAA_08701	-1.69	8.60E-07	subtilisin-like serine protease precursor
19264329	MAA_10043	-1.49	3.54E-16	destruxin synthetase, DtxS1
19256971	MAA_02685	-1.44	9.32E-09	chitinase
19261227	MAA_06941	-1.35	6.29E-03	extracellular serine-rich protein
19261280	MAA_06994	-1.33	6.14E-04	secretory lipase
19255482	MAA_01196	-1.32	2.84E-08	cysteine-rich secreted protein
19262103	MAA_07817	-1.31	4.09E-04	glycoside hydrolase family 76 protein
19261083	MAA_06797	-1.24	1.80E-07	peptidase family M28
19263922	MAA_09636	-1.21	3.14E-04	subtilisin-like protease
19263848	MAA_09562	-1.18	2.15E-02	fatty acid hydroxylase superfamily protein
19255800	MAA_01514	-1.18	5.15E-06	Peptidase A1
19255594	MAA_01308	-1.14	8.18E-06	Peptidase S8/S53, subtilisin/kexin/sedolisin
19263915	MAA_09629	-1.08	1.42E-06	Glycoside hydrolase, family 35
19260432	MAA_06146	-1.06	6.92E-10	glycoside hydrolase family 3 protein
19262454	MAA_08168	-1.03	8.43E-03	Peptidase S8, subtilisin-related protein
19255412	MAA_01126	-1.02	4.99E-04	Alpha/beta hydrolase fold-3
19262692	MAA_08406	1.10	1.22E-03	glycoside hydrolase family 16
19260689	MAA_06403	1.21	9.41E-06	glycoside hydrolase family 76 protein
19264818	MAA_10532	1.23	2.06E-02	peptidase family M3
19257096	MAA_02810	1.24	2.14E-03	Fungal chitosanase
19254778	MAA_00492	1.28	1.05E-11	subtilisin-like protease
19258326	MAA_04040	1.41	3.83E-03	alpha/beta hydrolase
19264734	MAA_10448	1.92	1.87E-05	hydrolase
19257622	MAA_03336	1.94	2.40E-07	Peptidase S8/S53, subtilisin/kexin/sedolisin
**Involved in cell wall composition, hydrocarbon epitopes and response to host immunnity**				
19263490	MAA_09204	-5.66	1.11E-18	Concanavalin A-like lectin/glucanase
19262598	MAA_08312	-5.18	1.21E-43	cell wall protein
19260495	MAA_06209	-3.07	2.13E-10	Concanavalin A-like lectin/glucanase
19255921	MAA_01635	-2.92	3.44E-04	integral membrane protein pth11
19262575	MAA_08289	-2.35	1.96E-03	cell surface protein (Mas1)
19256825	MAA_02539	-1.86	6.22E-06	cell surface protein (Mas1)
19261096	MAA_06810	-1.81	1.48E-06	FK506 suppressor Sfk1
19257628	MAA_03342	-1.53	2.32E-05	integral membrane protein
19256950	MAA_02664	-1.40	1.74E-07	actin filament organization protein App1-like protein
19256461	MAA_02175	-1.36	1.09E-04	immunoglobulin I-set domain-containing protein
19261596	MAA_07310	-1.34	5.44E-04	integral membrane family protein
19261473	MAA_07187	-1.09	1.24E-11	expression library immunization antigen 1
19264584	MAA_10298	1.04	2.25E-02	hydrophobin
19263358	MAA_09072	1.17	1.39E-02	cell wall galactomannoprotein Mp2/allergen F17-like protein
19255705	MAA_01419	4.01	2.64E-03	Calcium-mediated lectin
**Involved in cellular transport and homeostasis**				
19262600	MAA_08314	-5.35	1.77E-03	ATPase

19263823	MAA_09537	-5.23	1.69E-04	MFS1 family protein
19264334	MAA_10048	-3.21	7.70E-27	abc transporter
19264333	MAA_10047	-3.17	1.21E-25	V-type ATPase
19259842	MAA_05556	-2.72	2.47E-03	potassium/sodium efflux P-type ATPase
19259759	MAA_05473	-2.32	7.02E-05	siderophore iron transporter mirB
19262202	MAA_07916	-2.26	2.10E-02	major facilitator superfamily transporter
19256668	MAA_02382	-1.91	3.05E-09	Oligopeptide transporter OPT superfamily
19260674	MAA_06388	-1.90	3.36E-12	Cd2+/Zn2+ transporter protein
19258334	MAA_04048	-1.90	1.77E-03	major facilitator superfamily transporter
19264673	MAA_10387	-1.89	4.30E-06	ZIP Zinc transporter
19259726	MAA_05440	-1.83	7.89E-08	Phosphate transporter
19263051	MAA_08765	-1.78	7.74E-16	sugar transporter-like protein
19256663	MAA_02377	-1.71	2.94E-06	ATP-binding cassette transporter ifT1
19264281	MAA_09995	-1.63	9.51E-07	major facilitator superfamily transporter
19260324	MAA_06038	-1.57	2.17E-05	pH regulatory protein mrPacC
19261285	MAA_06999	-1.56	2.04E-03	major facilitator superfamily transporter
19256624	MAA_02338	-1.55	9.31E-07	General substrate transporter
19259755	MAA_05469	-1.46	7.73E-06	siderophore iron transporter
19264010	MAA_09724	-1.37	4.85E-04	ATPase, P-type, potassium/sodium efflux, fungal
19260235	MAA_05949	-1.31	3.63E-07	cation transporter
19263703	MAA_09417	-1.30	1.85E-06	oligopeptide transporter
19254593	MAA_00307	-1.30	1.74E-04	siderophore iron transporter
19255487	MAA_01201	-1.20	1.16E-03	Ctr copper transporter
19257045	MAA_02759	-1.18	3.29E-06	Ca2+ permeable channel
19264416	MAA_10130	-1.09	1.29E-02	Major facilitator superfamily domain, general substrate transporter
19259102	MAA_04816	-1.08	1.97E-07	cation transporting ATPase
19257500	MAA_03214	-1.08	2.47E-05	Amino acid transporter, transmembrane
19260619	MAA_06333	-1.05	1.43E-02	AAA family ATPase
19256077	MAA_01791	-1.05	2.89E-02	Major facilitator superfamily domain protein
19255636	MAA_01350	-1.02	1.05E-02	general substrate transporter
19260940	MAA_06654	-1.00	2.57E-11	ferrioxamine B transporter
19261806	MAA_07520	1.00	2.23E-03	major facilitator superfamily transporter
19262654	MAA_08368	1.01	1.85E-04	major facilitator superfamily transporter
19263316	MAA_09030	1.02	1.81E-02	Sodium/solute symporter
19263390	MAA_09104	1.10	1.93E-10	aaa ATPase
19260584	MAA_06298	1.19	1.72E-04	MFS transporter [Metarhizium robertsii]
19259936	MAA_05650	1.30	3.82E-02	MFS transporter
19258027	MAA_03741	1.57	1.62E-02	Major facilitator superfamily domain, general substrate transporter
19262905	MAA_08619	1.97	5.09E-07	choline transport protein
19256607	MAA_02321	2.02	8.58E-12	Major facilitator superfamily domain, general substrate transporter
19264481	MAA_10195	2.07	1.54E-04	Ctr copper transporter
19257555	MAA_03269	5.05	3.68E-02	membrane transporter
**Involved in transcription**				
19261383	MAA_07097	-3.19	5.20E-03	C2H2 transcription factor
19261778	MAA_07492	-1.78	3.68E-03	c6 zinc finger domain containing protein
19264428	MAA_10142	-1.64	9.04E-03	Zn(2)-C6 fungal-type DNA-binding domain protein
19264998	MAA_10714	-1.63	1.95E-03	C6 transcription factor
19257988	MAA_03702	-1.61	1.80E-04	transcription factor RfeG
19255066	MAA_00780	-1.47	3.20E-05	zinc finger protein dhhc domain containing protein
19258825	MAA_04539	-1.41	1.30E-05	Zn(2)-Cys(6) zinc finger domain protein
19256488	MAA_02202	-1.26	1.95E-08	zinc finger protein 58
19264231	MAA_09945	-1.22	2.95E-02	c6 zinc finger domain containing protein
19257739	MAA_03453	-1.21	2.18E-05	fungal specific transcription factor
19257577	MAA_03291	-1.13	7.12E-04	Zn(2)-C6 fungal-type DNA-binding domain protein
19255138	MAA_00852	-1.06	1.30E-02	swim zinc finger domain protein
19260351	MAA_06065	-1.06	2.47E-02	Basic-leucine zipper (bZIP) transcription factor
23633238	MAA_11790	1.02	1.80E-03	Zn(2)-Cys(6) zinc finger domain protein, partial
19264907	MAA_10621	1.04	1.64E-02	MYB DNA-binding domain-containing protein
19263417	MAA_09131	1.04	7.14E-06	zinc finger domain-containing protein
19262889	MAA_08603	1.04	4.53E-06	transcription factor Cys6
23633159	MAA_11711	1.04	1.09E-03	zinc finger domain-containing protein

23633188	MAA_11740	1.10	3.74E-14	Zn(2)-C6 fungal-type DNA-binding domain protein
19260311	MAA_06025	2.51	1.02E-12	PRO1A C6 Zink-finger protein
19264326	MAA_10040	2.84	2.30E-11	Zn(2)-Cys(6) zinc finger domain protein
19264403	MAA_10117	10.70	1.17E-02	Zn(2)-C6 fungal-type DNA-binding domain protein
**Involved in posttranslational modifications**				
23633036	MAA_11588	-9.96	3.33E-04	SET domain protein
19257561	MAA_03275	-3.64	3.05E-02	transferase
19255287	MAA_01001	-1.61	9.33E-05	GCN5-related N-acetyltransferase (GNAT) domain protein
19259756	MAA_05470	-1.30	3.09E-02	transferase family protein
19258535	MAA_04249	-1.16	1.25E-02	methyltransferase
19260739	MAA_06453	-1.04	2.93E-03	GCN5-related N-acetyltransferase (GNAT) domain protein
23632270	MAA_10821	-1.03	5.64E-03	ubiquitin-conjugating enzyme
19256214	MAA_01928	-1.02	4.42E-02	Ubiquitin 3 binding protein But2
19255584	MAA_01298	1.01	1.81E-02	O-methyltransferase
19260841	MAA_06555	1.02	2.03E-03	methyltransferase
19255392	MAA_01106	1.07	1.30E-08	Ubiquitin supergroup
19254406	MAA_00120	1.45	7.43E-18	Methyltransferase type 11
19262652	MAA_08366	1.80	3.44E-07	methyltransferase
**Involved in RNA/DNA processing and events**				
19254709	MAA_00423	-16.96	1.99E-21	Ribonuclease H-like protein
23632372	MAA_10923	-14.77	4.07E-09	Ankyrin repeat-containing domain protein
19254669	MAA_00383	-13.98	9.65E-10	Ribonuclease H-like protein
19255569	MAA_01283	-12.64	1.02E-07	Ribonuclease H-like protein
19263074	MAA_08788	-3.60	2.98E-05	ankyrin repeat-containing protein
19262592	MAA_08306	-3.45	1.96E-04	Ankyrin repeat protein
23632897	MAA_11449	-2.96	2.28E-17	Ankyrin repeat-containing domain protein
19257630	MAA_03344	-2.92	1.61E-02	ankyrin repeat containing protein
23632640	MAA_11192	-2.20	4.26E-03	ankyrin repeat protein [Metarhizium robertsii]
19262060	MAA_07774	-2.19	1.16E-02	ankyrin protein
19255215	MAA_00929	-2.17	3.22E-07	Ribonuclease H-like protein
19264518	MAA_10232	-1.88	2.63E-03	ankyrin repeat containing protein
19261648	MAA_07362	-1.85	9.81E-04	RNA-directed RNA polymerase (Sad-1)
19262660	MAA_08374	-1.34	9.35E-03	ankyrin repeat protein
19254739	MAA_00453	-1.22	3.61E-04	Ribonuclease T2, active site protein
19263485	MAA_09199	-1.12	4.92E-04	ATP-dependent RNA helicase DED1
19263684	MAA_09398	-1.10	5.56E-03	Ankyrin repeat protein
19262532	MAA_08246	-1.09	1.42E-04	ankyrin repeat domain containing protein
19255698	MAA_01412	-1.06	5.14E-04	Ankyrin repeat-containing domain protein
19258332	MAA_04046	-1.02	1.34E-02	RNA recognition motif domain protein
19263400	MAA_09114	1.01	5.60E-05	Ribonuclease H-like protein
23633286	MAA_11838	1.04	5.78E-05	Ankyrin repeat-containing domain protein
19255677	MAA_01391	1.06	1.23E-04	transposase
23632365	MAA_10916	1.06	1.38E-02	Ribonuclease H-like protein
19254667	MAA_00381	1.11	1.81E-02	Ribonuclease H-like protein
19264434	MAA_10148	1.13	4.59E-03	ATP-dependent DNA helicase PIF1
19264450	MAA_10164	1.14	7.77E-09	reverse transcriptase, RNaseH
19255217	MAA_00931	1.16	5.94E-12	Ribonuclease H-like protein
19264446	MAA_10160	1.20	8.86E-16	transposase-like protein
19264834	MAA_10548	1.23	3.44E-04	ATP-dependent DNA helicase PIF1
19264445	MAA_10159	1.24	5.66E-08	restless-like transposase
19259838	MAA_05552	1.24	7.29E-03	ribosomal protein L1
19264635	MAA_10349	1.28	6.35E-16	ATP-dependent DNA helicase PIF1
23632921	MAA_11473	1.30	2.14E-03	Ankyrin repeat-containing domain protein
23632196	MAA_10747	1.39	3.28E-03	ankyrin repeat protein
19264853	MAA_10567	1.42	3.77E-04	restless-like transposase
19264448	MAA_10162	1.50	1.25E-10	transposase-like protein
19255201	MAA_00915	1.66	6.20E-06	Ribonuclease H-like protein
19254647	MAA_00361	1.71	7.92E-15	transposase-like protein
23633210	MAA_11762	1.93	5.16E-04	reverse transcriptase
